# Discovery of Nine Dipeptidyl Peptidase-4 Inhibitors from *Coptis chinensis* Using Virtual Screening, Bioactivity Evaluation, and Binding Studies

**DOI:** 10.3390/molecules29102304

**Published:** 2024-05-14

**Authors:** Zixi Zhao, Ruonan Ma, Yuqing Ma, Liqiang Zhao, Lele Wang, Yuzhen Fang, Yuxin Zhang, Xia Wu, Xing Wang

**Affiliations:** 1 School of Traditional Chinese Medicine, Capital Medical University, Fengtai District, Beijing 100069, China; zhaozixi@mail.ccmu.edu.cn (Z.Z.); maruonan@mail.ccmu.edu.cn (R.M.); mayuqing@mail.ccmu.edu.cn (Y.M.); zlq0615@mail.ccmu.edu.cn (L.Z.); 2 School of Pharmacy, Minzu University of China, Haidian District, Beijing 100081, China; wanglele@muc.edu.cn (L.W.); fangyuzhen@muc.edu.cn (Y.F.)

**Keywords:** natural DPP-4 inhibitors, *Coptis chinensis*, enzyme inhibitory activity, molecular dynamics, surface plasmon resonance

## Abstract

The objective of this study was to identify multiple alkaloids in *Coptis chinensis* that demonstrate inhibitory activity against DPP-4 and systematically evaluate their activity and binding characteristics. A combined strategy that included molecular docking, a DPP-4 inhibition assay, surface plasmon resonance (SPR), and a molecular dynamics simulation technique was employed. The results showed that nine alkaloids in *Coptis chinensis* directly inhibited DPP-4, with IC_50_ values of 3.44–53.73 μM. SPR-based binding studies revealed that these alkaloids display rapid binding and dissociation characteristics when interacting with DPP-4, with K_D_ values ranging from 8.11 to 29.97 μM. A molecular dynamics analysis revealed that equilibrium was rapidly reached by nine DPP-4–ligand systems with minimal fluctuations, while binding free energy calculations showed that the ∆G_bind_ values for the nine test compounds ranged from −31.84 to −16.06 kcal/mol. The most important forces for the binding of these alkaloids with DPP-4 are electrostatic interactions and van der Waals forces. Various important amino acid residues, such as Arg125, His126, Phe357, Arg358, and Tyr547, were involved in the inhibition of DPP-4 by the compounds, revealing a mechanistic basis for the further optimization of these alkaloids as DPP-4 inhibitors. This study confirmed nine alkaloids as direct inhibitors of DPP-4 and characterized their binding features, thereby providing a basis for further research and development on novel DPP-4 inhibitors.

## 1. Introduction

Dipeptidyl peptidase-4 (DPP-4) is an important target for the clinical treatment of several diseases including diabetes, obesity, cardiovascular diseases, and non-alcoholic fatty liver disease (NAFLD) [1,2,3,4,5,6]. As a serine protease on the surface of cells, DPP-4 plays a role in the breakdown of peptides, including incretin hormones such as glucagon-like peptide-1 (GLP-1) and glucose-dependent insulinotropic polypeptide (GIP) [7]. The inhibition of DPP-4 can prolong the half-lives of these peptides, leading to increased insulin secretion and improved glucose control. DPP-4 inhibitors have been developed as a class of antidiabetic drugs and are used in the treatment of type 2 diabetes [8]. By inhibiting DPP-4, these drugs can enhance the action of GLP-1 and GIP, resulting in better blood sugar control. DPP-4 inhibitors are generally well tolerated and have a low risk of hypoglycemia compared to other antidiabetic drugs. Overall, the clinical importance of DPP-4 as a target lies in its involvement in glucose homeostasis and the potential therapeutic applications of DPP-4 inhibitors in diabetes and other related diseases. Therefore, inhibitors targeting DPP-4 have broad therapeutic potential for a variety of diseases.

**Figure 1:**
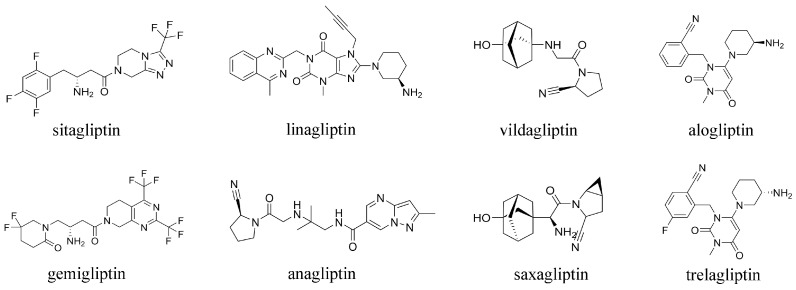
The structures of some DPP-4 inhibitors that are commercially available worldwide.

At present, a variety of DPP-4 inhibitors, such as sitagliptin, vildagliptin, saxagliptin, alogliptin, linagliptin, gemigliptin, trelagliptin, and anagliptin, are marketed worldwide (Figure 1). These DPP-4 inhibitors have the advantages of definite hypoglycemic effects, a low risk of hypoglycemia, no weight gain, good cardiovascular safety, and once-daily administration [9,10,11,12,13,14,15]. However, some potential side effects are also associated with them, such as nasopharyngitis, headache, upper respiratory tract infection, and mild gastrointestinal discomfort. Some rare adverse reactions include acute pancreatitis, arthralgia, heart failure, angioneurotic edema, hypersensitivity, elevated liver enzymes, diarrhea, cough, and a decreased absolute lymphocyte count [16,17,18,19,20,21]. In addition, the dosage of some DPP-4 inhibitors needs to be adjusted according to renal function; the exception is linagliptin, which is mainly excreted through the intestine while the others are mainly excreted through the kidney [22,23,24].

Compared with chemically synthesized drugs, natural herbal medicines are considered to have higher effectiveness and fewer side effects [25,26]. Therefore, an urgent need for novel DPP-4 inhibitors with good efficacy and safety in natural herbal medicines is recognized. *Coptis chinensis* is one of the earliest recognized and most valuable medicinal plants in China; it has been used to treat diabetes for over two thousand years. It has been shown to have various effects, such as lowering blood glucose, improving insulin secretion, and modulating intestinal microbiota [27,28,29,30]. Recent clinical studies have confirmed the efficacy of berberine (the main component of *Coptis chinensis*) for diabetes as it can induce insulin secretion in a glucose-dependent manner, thus avoiding the risk of hypoglycemia [31,32,33]. Therefore, it may be meaningful to screen and validate DPP-4 inhibitors from *Coptis chinensis* as they may offer new options for the development of effective and safe antidiabetic drugs.

**Figure 2:**
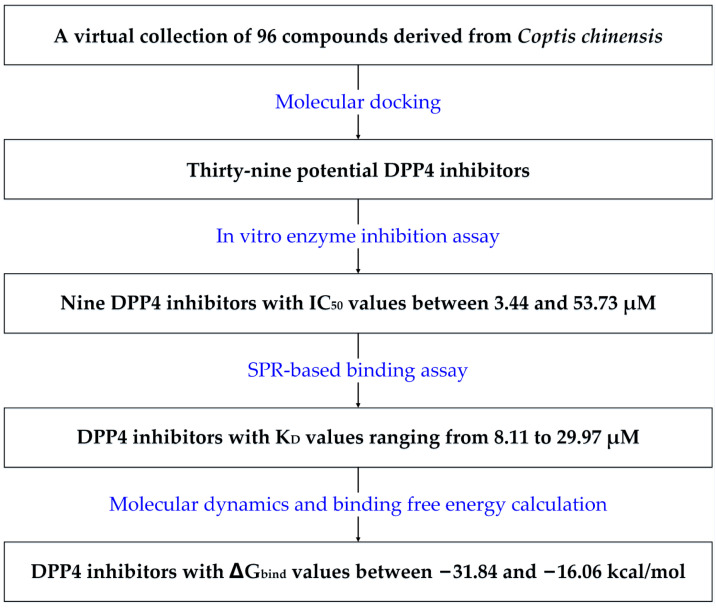
A schematic illustration of the discovery of DPP-4 inhibitors from *Coptis chinensis*.

This study accomplished the discovery and characterization of DPP-4 inhibitors from components of *Coptis chinensis* through an integrated approach that combined computational and experimental methods (Figure 2). Initially, molecular docking was performed to identify compounds with the potential to bind with DPP-4. Subsequently, an enzyme inhibition assay was used to validate the inhibitory activity of these potential compounds. Then, the SPR technique was employed to evaluate the affinity of the novel DPP-4 inhibitors for the DPP-4 protein and their binding–dissociation characteristics. Finally, a molecular dynamics simulation technique was used to analyze the interaction between the inhibitors and DPP-4. Natural DPP-4 inhibitors from *Coptis chinensis* were discovered in this work, which will facilitate the development of safe and effective DPP-4 inhibitors and reveal active ingredients in *Coptis chinensis* for treating diabetes.

## 2. Results

### 2.1. Potential DPP-4 Inhibitors Identified through Molecular Docking

**Figure 3:**
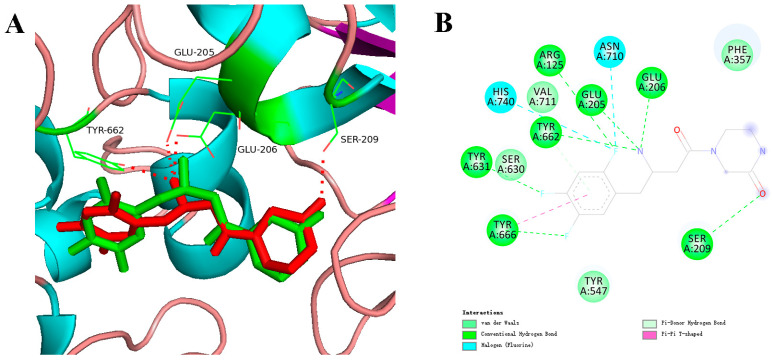
The interface (**A**) and interactions (**B**) between DPP-4 and ligand 8O3. Green and red sticks represent re-docked and co-crystallized conformations of 8O3, respectively. Red dotted lines indicate hydrogen bond interactions between ligands and DPP-4.

The reliability of the docking program was confirmed by re-docking 8O3 into the active site of DPP-4 from the co-crystal structure. A total score of 6.25 was achieved for DPP-4-8O3, and an RMSD of 0.71 Å was observed between the co-crystallized and re-docked conformations of 8O3 (Figure 3). This suggests that the experimentally observed binding mode was accurately reproduced by the docking program as the docked conformation was highly similar to the co-crystallized conformation. The amino acids Arg125, Glu205, Glu206, Ser209, Tyr631, Tyr662, Tyr666, Asn710, and His740 played a critical role in inhibiting DPP-4 activity at the 8O3 binding site. A screening of 96 natural compounds for DPP-4 inhibitors was subsequently conducted using docking procedures, resulting in the identification of 39 hits with docking scores exceeding 6.00, which is the score for the re-docking of 8O3 with DPP-4, indicating that the compounds have a strong affinity for the target protein. Inhibitory activity against DPP-4 was then evaluated in vitro for these hits.

### 2.2. In Vitro Evaluation of DPP-4 Inhibitory Activity

**Figure 4:**
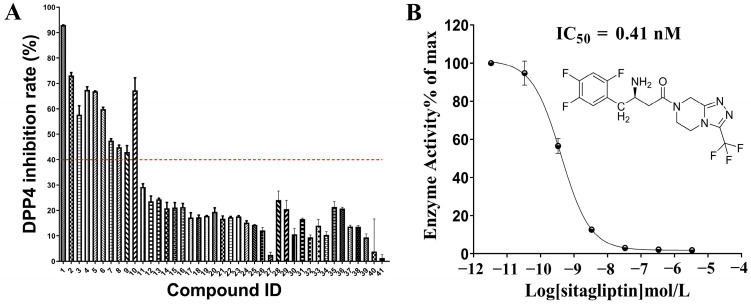
The initial screening results of the inhibitory activity of 39 compounds against DPP-4 (**A**) and the DPP-4 inhibitory activity of sitagliptin (**B**). In (**A**), the compounds represented by Compound IDs 1–40 are sitagliptin (CAS:486460-32-6), columbamine (CAS:3621-36-1), demethyleneberberine (CAS:25459-91-0), epiberberine (CAS:6873-09-2), groenlandicine (CAS:38691-95-1), coptisine (CAS:3486-66-6), berberine (CAS:2086-83-1), palmatine (CAS:3486-67-7), jatrorrhizine (CAS:3621-38-3), berberrubine (CAS:15401-69-1), ethyl caffeate (CAS:102-37-4), picrotin (CAS:21416-53-5), picroside ii (CAS:39012-20-9), protocatechuic acid methyl ester (CAS:2150-43-8), picroside i (CAS:27409-30-9), canadine (CAS:522-97-4), catalpol (CAS:2415-24-9), spongouridine (CAS:3083-77-0), caffeic acid dimethyl ether (CAS:2316-26-9), obacunone (CAS:751-03-1), isovanilline (CAS:621-59-0), D−mannoheptulose (CAS:3615-44-9), androsin (CAS:531−28−2), limonin (CAS:1180-71-8), N−trans−feruloyltyramine (CAS:66648-43-9), gentisic acid (CAS:490-79-9), danshensu (CAS:76822-21-4), sophocarpine (CAS:6483-15-4), sophoridine (CAS:6882-68-4), rhombifoline (CAS:529-78-2), N−methylcytisine (CAS:486-86-2), cytisine (CAS:485-35-8), D−tetrahydropalmatine (CAS:3520-14-7), phellodendrine (CAS:6873-13-8), corypalmine (CAS:27313-86-6), dauricine (CAS:524-17-4), sinomenine (CAS:115-53-7), phellodendrine chloride (CAS:104112-82-5), berbamine (CAS:478-61-5), and oxyberberine (CAS:549-21-3), respectively. The purity of all test compounds is above 98%, and the blank solvent is represented by compound ID 41. The DPP-4 inhibition rate (%) was calculated using the following formula: (maximum fluorescence value—sample fluorescence value)/maximum fluorescence value × 100%. The red dotted line indicates the 40% DPP4 inhibition rate line. In this study, compounds with a DPP4 inhibition rate higher than 40% were selected for further dose-response relationship analysis.

**Figure 5:**
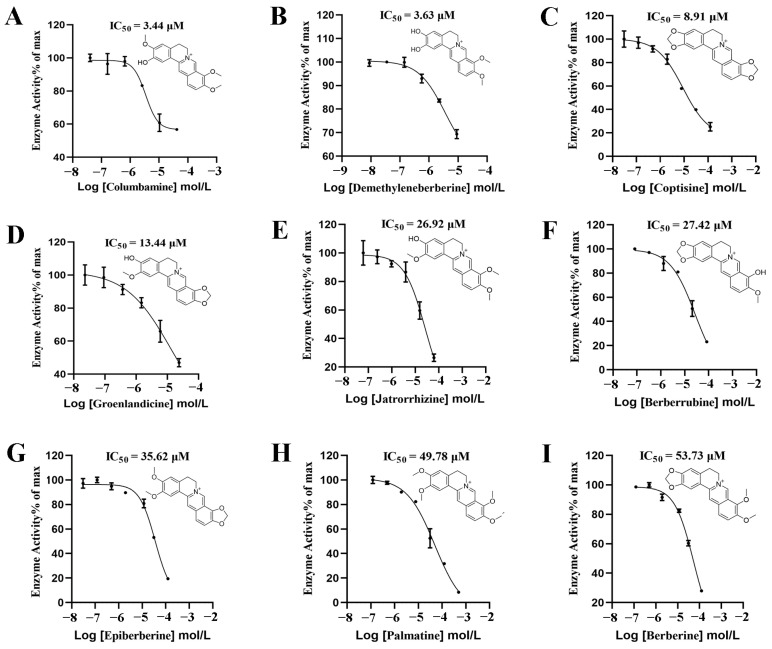
The DPP-4 inhibitory activity of columbamine (**A**), deMethyleneberberine (**B**), coptisine (**C**), groenlandicine (**D**), jatrorrhizine (**E**), berberrubine (**F**), epiberberine (**G**), palmatine (**H**), and berberine (**I**), measured using dose–response curves.

In a primary screening, 40% DPP-4 inhibition was exhibited by nine alkaloids in *Coptis chinensis*, namely columbamine, demethyleneberberine, epiberberine, groenlandicine, coptisine, berberine, palmatine, jatrorrhizine, and berberrubine, at a final concentration of 30 μM. Sitagliptin, which had an IC_50_ value of 0.41 nM (Figure 4), was used as a positive control. A further dose–response investigation revealed that nine alkaloids exhibited IC_50_ values of 3.44–53.73 μM for DPP-4 inhibition (Figure 5). Columbamine had the strongest inhibitory activity, with an IC_50_ value of 3.44 μM, while berberine had the weakest activity, with an IC_50_ value of 53.73 μM.

### 2.3. Interaction Analysis-Based Molecular Docking

**Figure 6:**
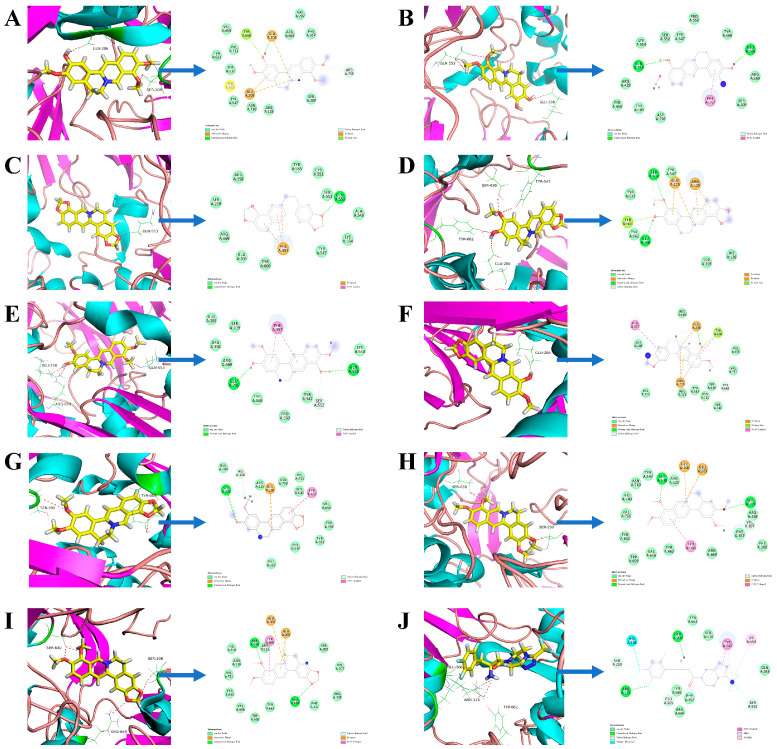
The interface of DPP-4 with various compounds including columbamine (**A**), deMethyleneberberine (**B**), coptisine (**C**), groenlandicine (**D**), Jatrorrhizine (**E**), berberrubine (**F**), epiberberine (**G**), palmatine (**H**), berberine (**I**), and sitagliptin (**J**) at the binding site.

Molecular docking was employed to investigate the binding sites between the ligands and DPP-4. The results showed that nine alkaloids had stable binding modes with DPP-4 (Figure 6). For instance, berberine formed two hydrogen bonds with the side chains of Ser630 and Arg669 and also established hydrophobic contacts with Glu205, Glu206, and Tyr666. Sitagliptin produced six hydrogen bonds with the side chains of Arg125, Glu205, Glu206, Ser209, and Ser552 and also had hydrophobic interactions with Tyr547 and Lys554. Some DPP-4 binding sites, including Glu205, Glu206, Tyr666, Phe357, and Arg125, can bind to at least four alkaloids, which may be a common characteristic of the interactions between these alkaloids and the DPP-4 protein. Some of the less frequently occurring amino acid residues, such as Arg358 for columbamine, Arg669 for berberine, and Lys554 and Tyr547 for sitagliptin, might be unique to the binding characteristics of these alkaloids.

### 2.4. Binding Analysis Based on SPR

**Figure 7:**
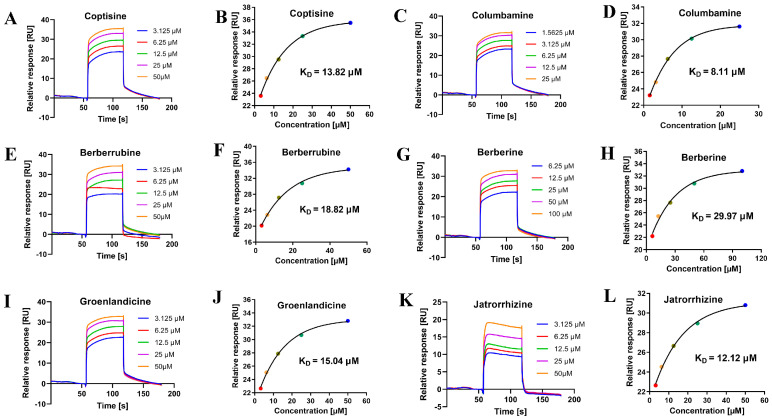
The sensorgrams and fitted curves for the binding and dissociation of DPP-4 with coptisine, columbamine, berberrubine, berberine, groenlandicine and jatrorrhizine (from (**A**) to (**L**)). The fitted curves were obtained using the ‘Affinity’ module of the evaluation software Biacore T200.

An SPR-based binding study showed that the test alkaloids bound to DPP-4 with fast binding and fast dissociation characteristics, and they had varying affinities, with K_D_ values ranging from 8.11 to 29.97 μM (Figure 7). The strongest affinity was observed for columbamine, with a K_D_ value of 8.11 μM, while berberine exhibited a relatively weak affinity, with a K_D_ value of 29.97 μM, which was consistent with the trend in its in vitro DPP-4 inhibitory activity.

### 2.5. Molecular Dynamics Simulation

**Figure 8:**
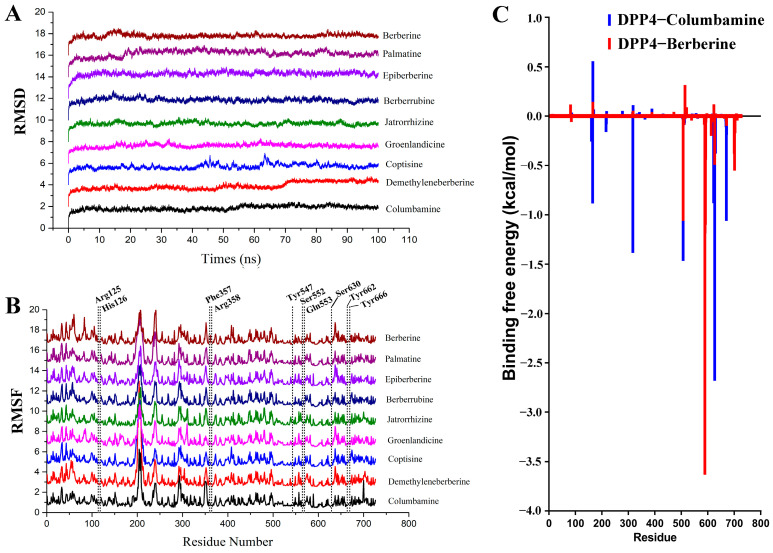
A molecular dynamics simulation analysis of DPP-4 and six of the test compounds. (**A**) The RMSD of the backbone atoms in the DPP-4–ligand system was calculated to evaluate the stability of the complex. The vertical offset was set to 2 in order to avoid overlap. (**B**) The RMSF values of the amino acid residues in the DPP-4–ligand system were computed to measure the flexibility of the protein. The key residues involved in binding are marked with dashed lines. The vertical offset was set to 2 in order to avoid overlap. (**C**) The MM/GBSA decomposition method was applied to estimate the contribution of each residue to the total binding free energies for the DPP-4–columbamine and DPP-4–berberine systems.

A molecular dynamics simulation was performed on the initial conformations of the DPP-4–inhibitor complexes obtained from the docking program. The RMSD values of the backbone atoms of DPP-4 relative to the initial structure were calculated to study the conformation dynamics of each system (Figure 8A). The results showed that the RMSD values of most compounds and DPP-4 complex system reached equilibrium quickly after 20 ns, with very low fluctuations (below 1 Å). However, coptisin and deMethyleneberberine reached equilibrium after 70 ns with very low fluctuations (below 1 Å). These results suggest that the protein conformation of DPP-4 could be bound and stabilized by the test compounds. Finally, the binding free energy and free energy decomposition of each system were calculated based on trajectory data from the last 20 ns.

The binding affinities of the nine DPP-4–ligand systems were calculated using MM/GBSA. The binding free energy (∆G_bind_) values for the nine test compounds ranged from −31.84 to −16.06 kcal/mol (Table 1). Hydrophobic, electrostatic, and hydrogen bonding interactions play important roles in the interactions between them. The hydrophobic groups of the nine alkaloids, which include multiple phenyl and methyl groups, interact with the hydrophobic region of the protein active pocket through amino acid residues such as Phe357, Tyr662, and Tyr666, thereby enhancing their affinity with the DPP-4 protein. Moreover, electrostatic interaction also plays a large role in the interaction between these nine alkaloids and DPP-4. The N atoms of the nine alkaloids, which are all quaternary ammonium alkaloids and carry positive charges, interact with a charged region in the protein active pocket, such as Glu205, Glu206, and other amino acid residues, increasing their affinity with the DPP-4 protein. The important structural basis for their DPP-4-inhibitory effect lies in the benzene ring, positively charged N atom, and hydroxyl group of these nine alkaloids, which enable them to bind firmly to DPP-4.

The root mean square fluctuation (RMSF) of each amino acid residue of the DPP-4 protein was calculated to investigate the binding stability of the nine test compounds with DPP-4 (Figure 8B). A similar RMSF distribution was observed for the nine systems overall. Lower RMSF values were exhibited by the key common amino acid residues (i.e., Arg125, His126, Phe357, Arg358, Tyr547, Ser552, Gln553, Ser630, Tyr662, and Tyr666) than the other regions of DPP-4, which means that a strong binding force was exerted by them toward DPP-4 and the tested compounds.

## 3. Discussion

Although previous studies in the literature reported that berberine, the main component of *Coptis chinensis*, demonstrates inhibitory activity against DPP-4, this study found that several other alkaloids in *Coptis chinensis* in addition to berberine can also inhibit DPP-4’s activity, and some of them demonstrate stronger activity than berberine. This discovery possesses innovative value and provides references for revealing the relationship between the DPP-4 inhibitory activity of isoquinoline alkaloids and their structures, providing new clues for the development of more effective DPP-4 inhibitors.

In the enzymatic reaction assay, the IC_50_ values of berberine were found to be close to those reported in the literature [34,35], and the other eight alkaloids all demonstrated stronger DPP-4 inhibitory activity than berberine. Using columbine and berberine as examples, the structures and activities of the alkaloids in *Coptis chinensis* were analyzed. Columbine has a hydroxyl and methoxy group at the same position as the methanedioxyl group of berberine, which allows columbine to form hydrogen bonds and hydrophobic interactions with the key amino acid residues (e.g., Glu206 and Tyr666) of the DPP-4 protein. These interactions enhance the binding affinity of columbine with the DPP-4 protein. Similar observations were made for demethyleneberberine, berberrubine, and jatrorrhizine. This phenomenon was confirmed by both SPR-based binding assays and molecular dynamics simulations. Columbamine has a stronger affinity and a lower binding free energy than berberine, indicating that the hydroxyl group on the isoquinoline core can enhance the affinity between alkaloids and DPP-4 protein, thereby improving its DPP-4 inhibitory activity (Table S1).

The material basis and mechanism of the use of *Coptis chinensis* in diabetes treatment are further expanded in this work. In addition to berberine, many kinds of alkaloids in *Coptis chinensis* also possess DPP-4 inhibitory activity, which suggests that *Coptis chinensis*’s hypoglycemic effect may be the result of the collective action of its various alkaloids. Numerous studies have reported that many alkaloids, including palmatine, columbamine, and coptisine, possess properties such as reducing blood sugar, promoting insulin secretion, improving insulin resistance, and enhancing glucose utilization [36,37,38,39,40,41]. A comprehensive improvement in streptozotocin-induced diabetic mice was observed when various alkaloids in *Coptis chinensis* were combined, as evidenced by lowered blood sugar levels, an increased insulin content, and an improved lipid metabolism [42]. A theoretical basis for the application of *Coptis chinensis* in the treatment of diabetes is provided by these findings. Although all these alkaloids have the ability to inhibit DPP-4, their differing chemical structures and binding characteristics suggest a potential synergistic role in diabetes treatment. The combination of these alkaloids in a drug formulation has the potential to enhance clinical efficacy and reduce adverse reactions and drug resistance, thereby offering new insights and directions for the development of diabetes drugs.

## 4. Materials and Methods

### 4.1. Molecular Docking-Based Virtual Screening

To identify DPP-4 inhibitors in *Coptis chinensis*, a virtual library of 96 compounds from *Coptis chinensis* was constructed for a docking-based virtual screening using Surflex-Dock, a semi-flexible docking program, in the Sybyl X-2.0 package (Tripos, Cincinnati, OH, USA). Surflex-Dock employs a unique empirical scoring function and a search engine based on molecular similarity to dock ligand molecules to protein binding sites. The compound library was downloaded from the NCBI PubChem database (https://www.ncbi.nlm.nih.gov/pccompound, accessed on 16 January 2024.) in 2D (sdf) format. The optimization of these compounds and the molecular docking process were carried out according to our previous work [43,44].

The target protein for docking is the A chain of the DPP-4 crystal structure (PDB ID: 5Y7H), which was resolved by X-ray crystallography at 3.00 Å resolution [45]. The preparation of the DPP-4 crystal structure involved the elimination of crystallographic water molecules and the addition of hydrogen atoms. Subsequently, an energy minimization process was conducted utilizing an AMBER7 F99 force field. This process was carried out in accordance with the standard parameters of the SYBYL X-2.0 software. The DPP-4 active site, located within the binding pocket, is defined as the area within a 0.5 Å distance from the recognized inhibitor 8O3, chemically known as (R)-4-(3-amino-4-(2,4,5-trifluorophenyl)butanoyl)piperazin-2-one, with the molecular formula C_14_H_16_F_3_N_3_O_2_ [45].

In order to evaluate the precision and robustness of the docking program, 8O3 was removed from the co-crystal structure and subsequently re-docked into the DPP-4 active site using the pre-established parameters of the Surflex-Dock docking program. The root mean square deviation (RMSD) was employed to measure the discrepancy between the co-crystallized and docked configurations of all heavy atoms in 8O3, as dictated by the subsequent formula [46]: “d” represents the distance between N pairs of corresponding atoms, with hydrogen atoms being the exception. A smaller RMSD value indicates a greater similarity between the co-crystal and docked configurations of 8O3. Diagrams of the protein–ligand contacts were generated using the show 2D Diagram program in Discovery Studio 3.0 software.

$$\mathrm{RMSD}=\sqrt{\frac{1}{N}\overset{i=N}{\underset{i=1}{\sum }}d_{i}^{2}}$$

### 4.2. DPP-4 Inhibition Assay

The DPP-4 inhibitory activity of 39 potential compounds obtained via virtual screening was tested using a commercial DPP-4 inhibitor screening assay kit (Item No. 700210, Cayman Chemical Company, Ann Arbor, MI, USA), and the kit’s instructions were followed exactly. The fluorescence intensity was measured at an excitation/emission wavelength of 360/460 nm using an EnVision Multilabel Reader (2104 PerkinElmer, Waltham, MA, USA). The possibility of false positives was eliminated by assessing the autofluorescence of each test compound. The test compounds, documented to be isolated from *Coptis chinensis* and structurally identified [47,48,49,50], were purchased from MCE (Medchem Express, Monmouth Junction, NJ, USA) and had a purity exceeding 98% (Figures S1–S18). These compounds were prepared at a concentration of 30 μM in deionized water that contained 0.5% DMSO. Sitagliptin (0.60 μM, Sigma-Aldrich, St. Louis, MO, USA) was used as a positive control, while deionized water containing 0.5% DMSO served as a negative control [51]. The half-maximal inhibitory concentration (IC_50_) was determined using GraphPad Prism 9 software.

### 4.3. SPR-Based Binding Assay

The binding affinity between DPP-4 and six alkaloids in *Coptis chinensis* was evaluated using the BIAcore T200 system (GE Healthcare, Uppsala, Sweden). All SPR-related materials were obtained from GE Healthcare, and DPP-4 (D3446) was purchased from Sigma-Aldrich Chemical Co. (St. Louis, MO, USA). The DPP-4 protein, dissolved in PBS at a concentration of 40 ng/μL, was immobilized onto the surface of a CM5 sensor chip using a standard amine-coupling procedure. The test compounds were prepared at varying concentrations in a solution of Tris-Hcl (pH 8.0) and injected over the surface of the chip at a rate of 30 μL·min^−1^ and a temperature of 25 °C. A reference flow cell without the immobilization of DPP-4 served as a control for non-specific binding. The association and dissociation kinetics of each test compound with DPP-4 were observed for a duration of 1 min. The chip could be regenerated and reused multiple times after it was treated with glycine–HCl (10 mM; pH 2.0). The test compounds were evaluated using the single-cycle kinetics mode across a two-fold concentration series (1.17–100 µM). The equilibrium constant (K_D_) was calculated based on the 1:1 reaction ratio between the molecule and protein, using the ‘affinity” module in the Biacore T200 software [52].

### 4.4. Molecular Dynamics and Binding Free Energy Calculation

A molecular dynamics simulation was applied to analyze the binding features and free energy of nine alkaloids in *Coptis chinensis* with the DPP-4 protein. A simulation system was set up which included a combination of the DPP-4 protein, a test compound, ions, and water, according to our previous work [43]. The initial atomic coordinates of the DPP-4-inhibitor complex were obtained using the Surflex-Dock method for the molecular dynamics simulation. Nine separate simulation boxes were created to enable the molecular dynamics simulation of nine compounds binding to DPP-4.

The geometry of the ligand was optimized using the B3LYP/6-31G* level of theory, while the formal charges were computed using the HF/6-31G* method in the program Gaussian 09 [53]. Partial charges were generated by fitting the electrostatic potentials with the Gaussian program, employing the restrained electrostatic potential (RESP) fitting technique found in the Amber20 package [54]. Each system’s water box was constructed with the TIP3P water model [55], and either chlorine or sodium ions were introduced to achieve system neutrality prior to the MD simulation. The DPP-4 protein and the ligands were assigned the ff14SB force field and the general Amber force field (GAFF), respectively, utilizing the LEaP module in the Amber20 package [56].

To achieve a low-energy starting conformation for the subsequent MD simulations, energy minimization was performed on each simulation box. Initially, the steepest descent method was employed with four thousand steps, followed by the conjugate gradient method with six thousand steps. The minimization process was carried out on the entire simulation system, which included the protein, ligand, ions, and water. Subsequently, the solutes, which consisted of the protein and ligand, were minimized. The system was heated from 0 to 300 K over a period of 300 ps using a Langevin thermostat system under a canonical ensemble and a harmonic restraint force constant set at 10.0 kcal mol^−1^ Å^−2^. The system was equilibrated for a duration of 10 ns under conditions of constant temperature and pressure (isothermal–isobaric), maintaining a pressure of 1.0 bar. The barostat bath’s relaxation time was established at 2.0 ps. Following this, the production simulation was carried out for 100 ns under isothermal–isobaric conditions, with periodic boundary conditions in place. A time step of 2 fs was chosen, and the SHAKE algorithm was utilized to constrain the bonds involving hydrogen atoms. The particle mesh Ewald method was employed to manage long-range electrostatic interactions [57]. For short-range interactions, a cut-off value of 10.0 Å was set.

The binding free energy for DPP-4 and each test compound was estimated using molecular mechanics energies and techniques such as generalized born and surface area continuum solvation [58]. The total binding free energy was further dissected into individual contributions from each amino acid residue, facilitating the identification of the most crucial residues in the binding of DPP-4 to each test compound using the MM/GBSA decomposition method [59].

### 4.5. Statistical Method

Statistical analysis and graphical representation were carried out using GraphPad Prism V9 software (GraphPad Software, San Diego, CA, USA). Data obtained from in vitro experiments were subjected to an ANOVA (one-way analysis of variance) and Dunnett’s multiple comparison test for analysis. A ***p value of less than 0.001 was considered significant.

## 5. Conclusions

Nine alkaloids from *Coptis chinensis* were discovered to be direct DPP-4 inhibitors were discovered from by employing a systematic strategy of computational simulation, biological verification, and binding studies. The anti-DPP-4 activity and binding characteristics of these compounds were evaluated using multiple methods. The characteristics of the binding between DPP-4 and the nine alkaloids present a valuable reference for future research to enhance their efficacy and selectivity via structural optimization, thereby laying the foundation for the development of DPP-4-targeting agents. Moreover, this study forms a basis and reference for understanding the material basis and mechanism underlying the therapeutic effect of *Coptis chinensis* on diabetes.

## Figures and Tables

**Table 1:** Binding free energies and intermolecular interaction energy components of systems of DPP-4 and nine alkaloids in *Coptis chinensis*, calculated by the MM/GBSA approach (kcal/mol). Here, the van der Waals and coulomb energies are denoted by E_vdw_ and E_ele_, respectively. The polar and non-polar solvation contributions, E_GB_ and E_SURF_, were determined by solving the GB equations. G_sol_ represents the total solvation free energy, while G_bind_ represents the binding energy between DPP-4 and the ligands. G_bind_ was approximated by ∆G_bind_ = G_com_ − (G_pro_ + G_lig_) ≈ ∆E_vdw_ + ∆E_ele_ + ∆G_sol_.

	Energy Component	Average	Std. Dev.	Std. Err. of Mean
DPP-4– Columbamine	ΔE_vdw_	−35.93	2.65	0.08
	∆E_ele_	−282.02	8.30	0.26
	∆E_GB_	291.16	7.77	0.25
	∆E_SURF_	−5.05	0.16	0.00
	∆G_gas_	−317.95	8.30	0.26
	∆G_sol_	286.11	7.77	0.25
	∆G_bind_	−31.84	2.54	0.08
DPP-4– Demethyleneberberine	ΔE_vdw_	−23.95	5.13	0.16
	∆E_ele_	−277.99	17.25	0.55
	∆E_GB_	276.92	16.17	0.51
	∆E_SURF_	−3.70	0.41	0.01
	∆G_gas_	−301.94	14.42	0.46
	∆G_sol_	273.21	16.45	0.52
	∆G_bind_	−28.73	5.45	0.17
DPP-4– Coptisine	ΔE_vdw_	−28.79	2.24	0.07
	∆E_ele_	−216.09	8.99	0.28
	∆E_GB_	229.81	8.74	0.28
	∆E_SURF_	−2.90	0.15	0.00
	∆G_gas_	−244.88	9.13	0.29
	∆G_sol_	226.91	8.70	0.28
	∆G_bind_	−17.98	2.15	0.07
DPP-4– Groenlandicine	ΔE_vdw_	−23.60	3.52	0.11
	∆E_ele_	−283.96	10.39	0.33
	∆E_GB_	291.75	10.14	0.32
	∆E_SURF_	−3.61	0.33	0.01
	∆G_gas_	−307.57	11.24	0.36
	∆G_sol_	288.15	9.98	0.32
	∆G_bind_	−19.42	3.45	0.11
DPP-4– Jatrorrhizine	ΔE_vdw_	−32.43	2.76	0.09
	∆E_ele_	−228.65	8.00	0.25
	∆E_GB_	238.54	7.04	0.22
	∆E_SURF_	−4.42	0.19	0.01
	∆G_gas_	−261.09	8.05	0.25
	∆G_sol_	234.11	7.03	0.22
	∆G_bind_	−26.97	2.89	0.09
DPP-4– Berberrubine	ΔE_vdw_	−32.80	3.32	0.11
	∆E_ele_	−289.40	10.88	0.34
	∆E_GB_	300.01	8.61	0.27
	∆E_SURF_	−4.55	0.23	0.01
	∆G_gas_	−322.21	10.99	0.35
	∆G_sol_	295.45	8.58	0.27
	∆G_bind_	−26.75	4.77	0.15
DPP-4– Epiberberine	ΔE_vdw_	−30.50	2.68	0.08
	∆E_ele_	−241.47	8.29	0.26
	∆E_GB_	260.01	8.56	0.27
	∆E_SURF_	−4.10	0.38	0.01
	∆G_gas_	−271.98	9.41	0.30
	∆G_sol_	255.92	8.38	0.26
	∆G_bind_	−16.06	2.56	0.08
DPP-4– Palmatine	ΔE_vdw_	−33.91	2.33	0.07
	∆E_ele_	−255.44	7.37	0.23
	∆E_GB_	269.54	7.02	0.22
	∆E_SURF_	−4.27	0.24	0.01
	∆G_gas_	−289.35	7.45	0.24
	∆G_sol_	265.27	6.98	0.22
	∆G_bind_	−24.08	2.58	0.08
DPP-4– Berberine	ΔE_vdw_	−30.70	2.31	0.07
	∆E_ele_	−204.52	12.53	0.40
	∆E_GB_	218.71	12.96	0.41
	∆E_SURF_	−3.20	0.36	0.01
	∆G_gas_	−235.23	12.68	0.40
	∆G_sol_	215.51	12.82	0.41
	∆G_bind_	−19.72	2.40	0.08

## Data Availability

Data are contained within the article.

## Supplementary Materials

The following supporting information can be downloaded at https://www.mdpi.com/article/10.3390/molecules29102304/s1, Figures S1–S18: The 1H NMR spectra and HPLC chromatograms of columbamine, demethyleneberberine, epiberberine, groenlandicine, coptisine, berberine, palmatine, jatrorrhizine, and berberrubine. Table S1. Summary of the information of the DPP-4 inhibitors discovered in this study.
