# Supplementary material for: Discovery of Nine Dipeptidyl Peptidase-4 Inhibitors from Coptis chinensis Using Virtual Screening, Bioactivity Evaluation, and Binding Studies
Source: Molecules. 2024 May 14;29(10):2304. doi: 10.3390/molecules29102304 (PMC11123979; doi:10.3390/molecules29102304)
Supplement: Supplementary file 1 [file molecules-29-02304-s001.zip › molecules-2977035-supplementary.pdf]

molecules

Supporting Information

Discovery of Nine Dipeptidyl Peptidase-4 Inhibitors from *Coptis chinensis* Using Virtual Screening, Bioactivity Evaluation, and Binding Studies

Zixi Zhao [1], Ruonan Ma [1], Yuqing Ma [1], Liqiang Zhao [1], Lele Wang [2], Yuzhen Fang [2], Yuxin Zhang [2,*], Xia Wu [1,*] and Xing Wang [1,*]

1 School of Traditional Chinese Medicine, Capital Medical University, Fengtai District, Beijing 100069, China; zhaozixi@mail.ccmu.edu.cn (Z.Z.); maruonan@mail.ccmu.edu.cn (R.M.); mayuqing@mail.ccmu.edu.cn (Y.M.); zlq0615@mail.ccmu.edu.cn (L.Z.)
2 School of Pharmacy, Minzu University of China, Haidian District, Beijing 100081, China; wanglele@muc.edu.cn (L.W.); fangyuzhen@muc.edu.cn (Y.F.)
* Correspondence: zhangyuxin@muc.edu.cn (Y.Z.); wuxia6710@ccmu.edu.cn (X.W.); wangxing@ccmu.edu.cn (X.W.); Tel.: +86-10-83911633 (Xing Wang); Fax: +86-10-83911627 (Xing Wang)

Contents

Figure S1. 1H NMR spectrum of columbamin sample (DMSO, 400 MHz).
Figure S2. HPLC chromatogram of columbamin sample.
Figure S3. 1H NMR spectrum of demethyleneberberine sample (DMSO, 400 MHz).
Figure S4. HPLC chromatogram of demethyleneberberine sample.
Figure S5. 1H NMR spectrum of epiberberine sample (DMSO, 400 MHz).
Figure S6. HPLC chromatogram of epiberberine sample.
Figure S7. 1H NMR spectrum of groenlandicine sample (DMSO, 400 MHz).
Figure S8. HPLC chromatogram of groenlandicine sample.
Figure S9. 1H NMR spectrum of coptisine sample (DMSO, 400 MHz).
Figure S10. HPLC chromatogram of coptisine sample.
Figure S11. 1H NMR spectrum of berberine sample (DMSO, 400 MHz).
Figure S12. HPLC chromatogram of berberine sample.
Figure S13. 1H NMR spectrum of palmatine sample (DMSO, 400 MHz).
Figure S14. HPLC chromatogram of palmatine sample.
Figure S15. 1H NMR spectrum of jatrorrhizine sample (DMSO, 400 MHz).
Figure S16. HPLC chromatogram of jatrorrhizine sample.
Figure S17. 1H NMR spectrum of berberrubine sample (DMSO, 400 MHz).
Figure S18. HPLC chromatogram of berberrubine sample.
Table S1. Summary of the information of the DPP-4 inhibitors discovered in this study.

Figure S1. 1H NMR spectrum of columbamin sample (DMSO, 400 MHz).

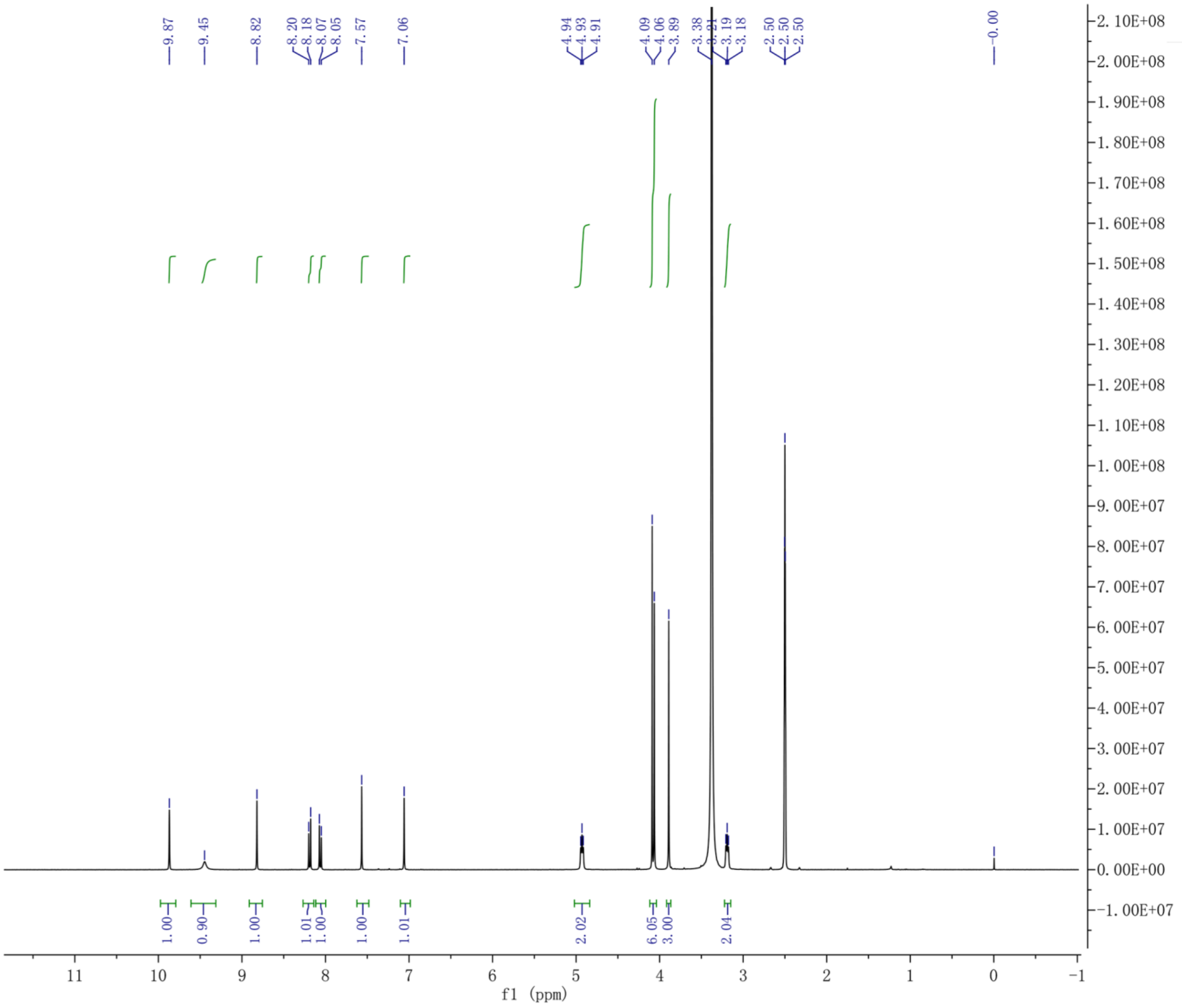

Figure S2. HPLC chromatogram of columbamin sample.

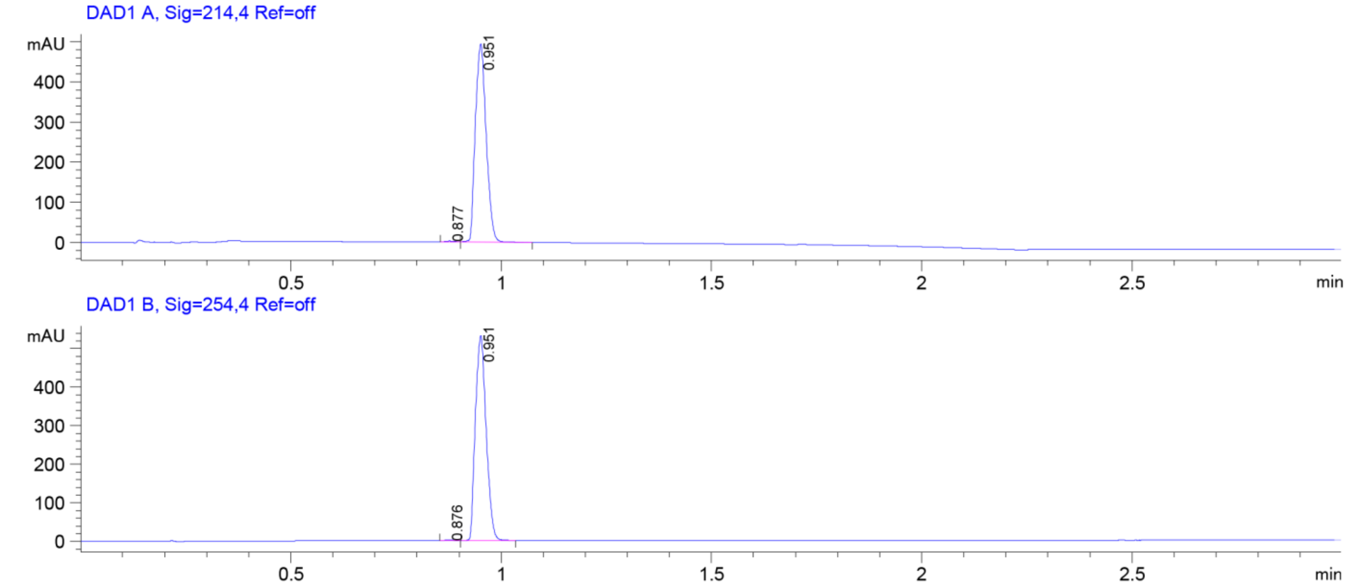

Integration Results for DAD1 A, Sig=214,4 Ref=off

RetTim	Width	Area	Height	Area%
0.88	0.02	2.65	2.00	0.30
0.95	0.03	874.97	492.36	99.70

Integration Results for DAD1 B, Sig=254,4 Ref=off

RetTim	Width	Area	Height	Area%
0.88	0.02	2.24	1.70	0.24
0.95	0.03	941.28	531.80	99.76

Figure S3. 1H NMR spectrum of demethyleneberberine sample (DMSO, 400 MHz).

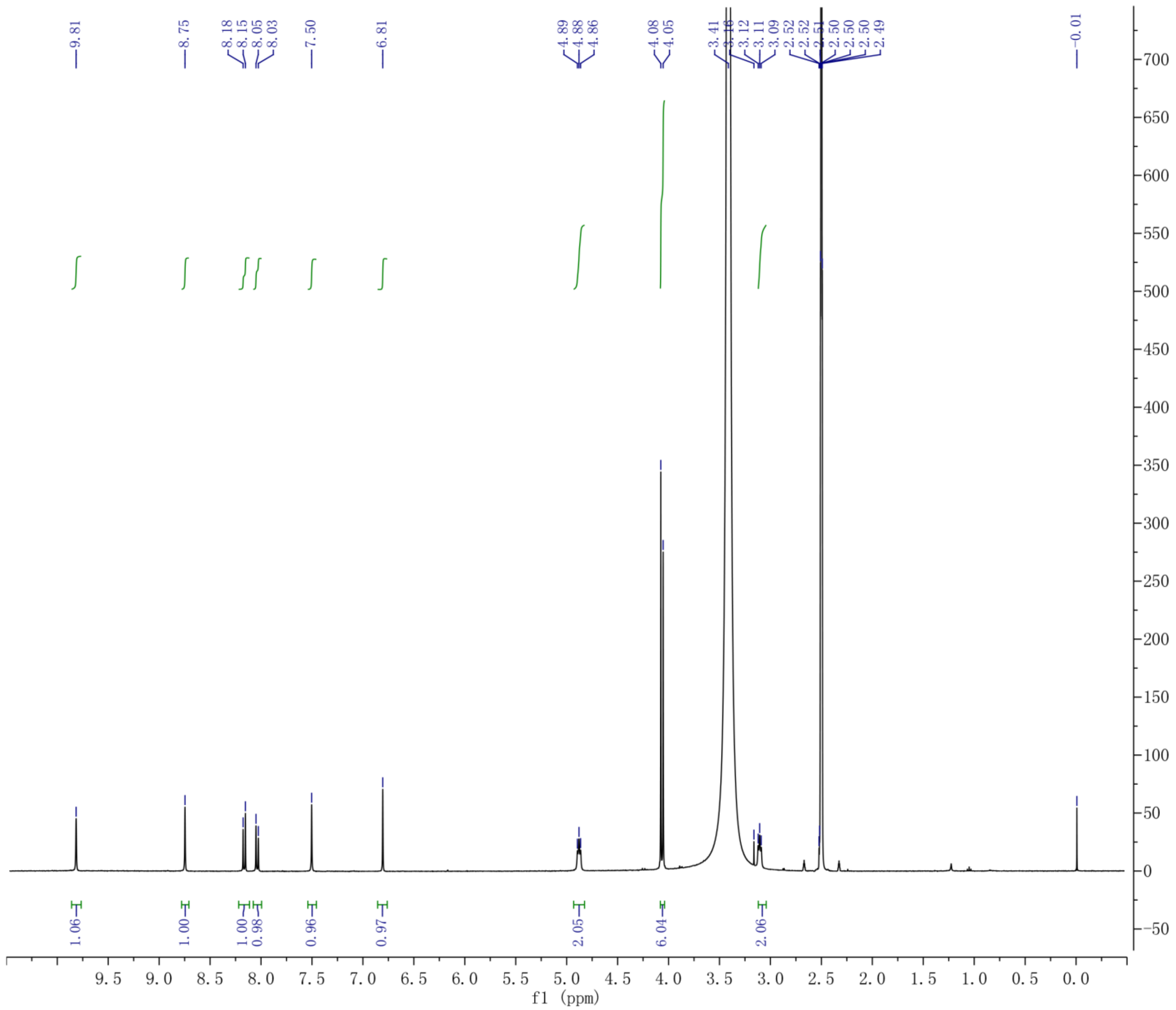

Figure S4. HPLC chromatogram of demethyleneberberine sample.

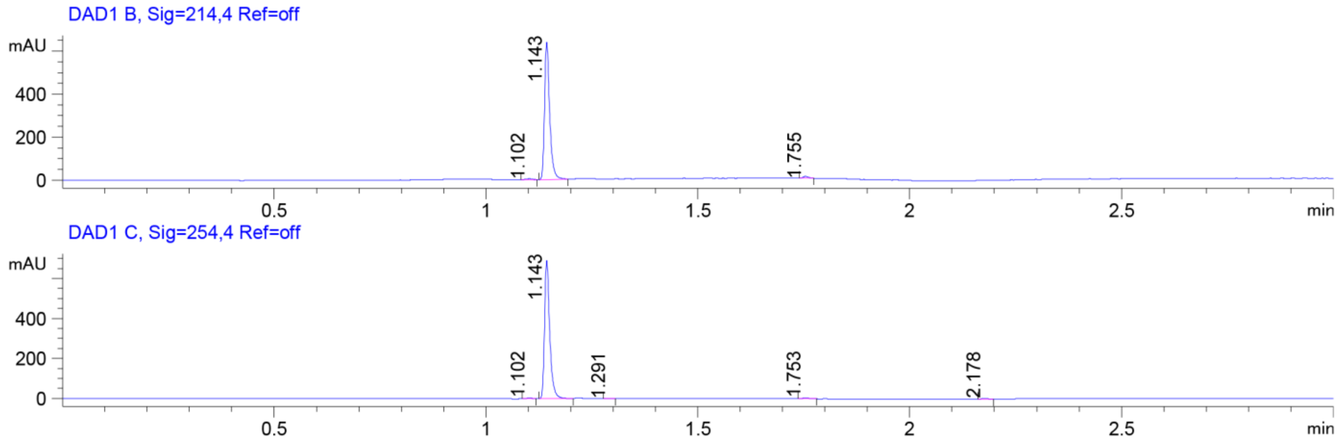

Integration Results for DAD1 B, Sig=214,4 Ref=off

RetTim	Width	Area	Height	Area%	MS(+)
1.10	0.02	4.22	4.41	0.76	310
1.14	0.01	542.35	640.64	98.09	324
1.75	0.01	6.34	7.83	1.15	283

Integration Results for DAD1 C, Sig=254,4 Ref=off

RetTim	Width	Area	Height	Area%	MS(+)
1.10	0.01	3.98	4.47	0.66	310
1.14	0.01	594.19	694.05	98.10	324
1.29	0.01	0.39	0.55	0.07	324
1.75	0.02	5.35	5.50	0.88	283
2.18	0.02	1.77	1.71	0.29	130

Figure S5. 1H NMR spectrum of epiberberine sample (DMSO, 400 MHz).

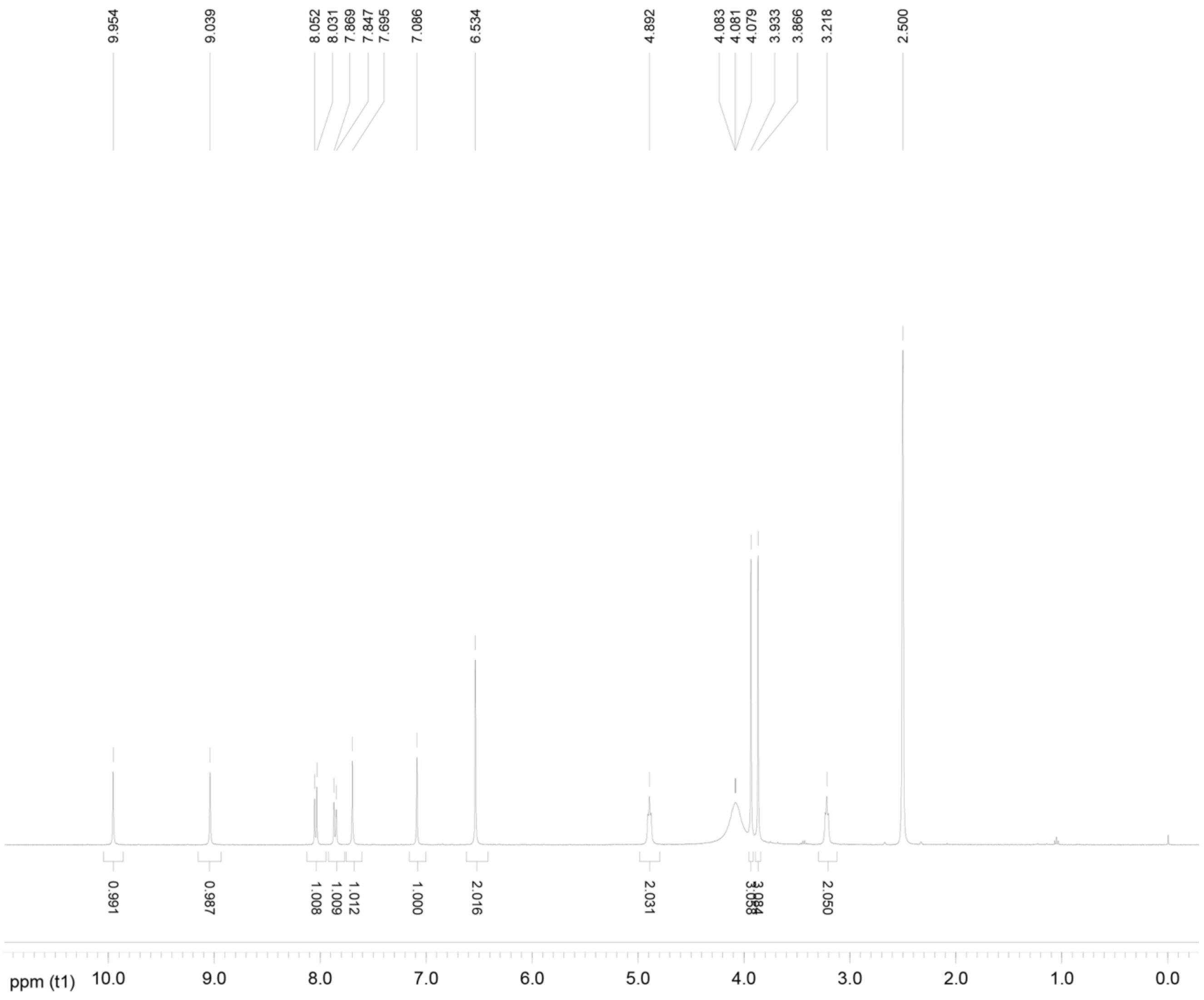

Figure S6. HPLC chromatogram of epiberberine sample.

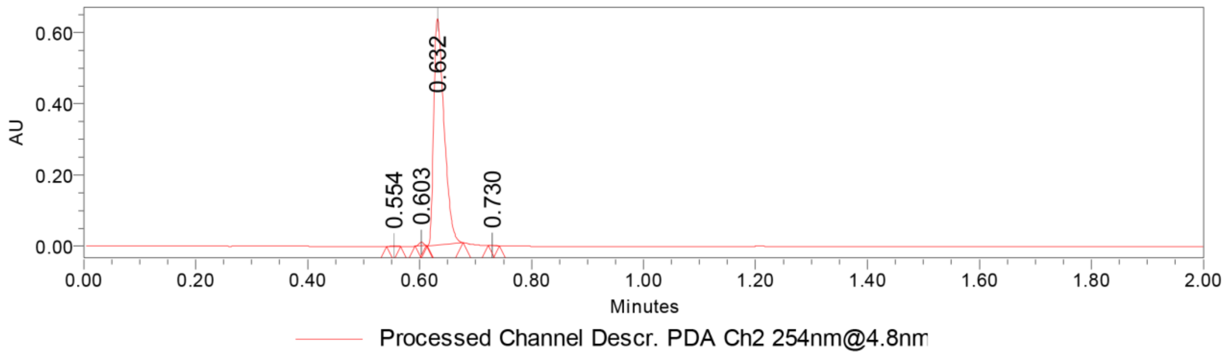

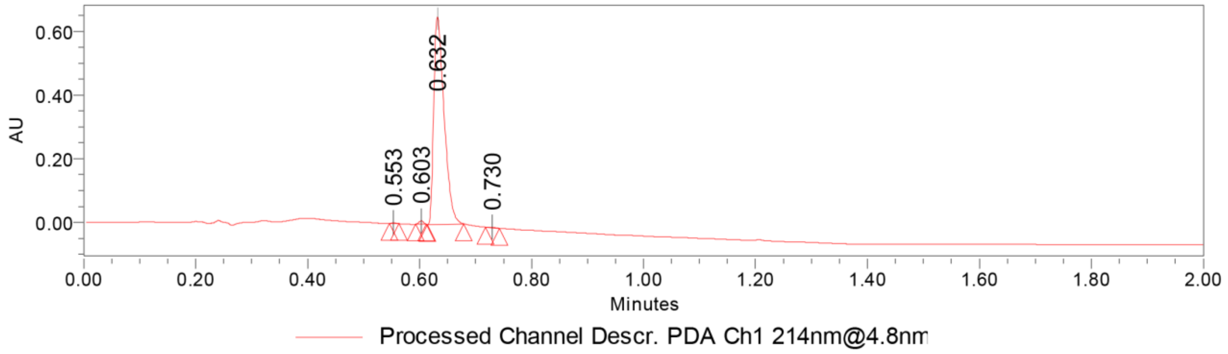

Peak Results
Channel Name: PDA Ch2 254nm@4.8nm

	RT	Width (sec)	Area	Height	% Area	Base Peak (Combined) (m/z)	Channel Name
1	0.554	1.450	1423	2123	0.17	337.14	PDA Ch2 254nm@4.8nm
2	0.603	1.150	5834	10176	0.71	337.16	PDA Ch2 254nm@4.8nm
3	0.632	3.800	811232	635590	99.02	322.07	PDA Ch2 254nm@4.8nm
4	0.730	1.200	741	1138	0.09	334.05	PDA Ch2 254nm@4.8nm

Peak Results
Channel Name: PDA Ch1 214nm@4.8nm

	RT	Width (sec)	Area	Height	% Area	Base Peak (Combined) (m/z)	Channel Name
1	0.553	1.050	1240	2122	0.15	150.89	PDA Ch1 214nm@4.8nm
2	0.603	1.100	6101	10747	0.72	337.16	PDA Ch1 214nm@4.8nm
3	0.632	3.900	835443	652198	99.03	322.07	PDA Ch1 214nm@4.8nm
4	0.730	1.500	822	1215	0.10	334.04	PDA Ch1 214nm@4.8nm

Figure S7. 1H NMR spectrum of groenlandicine sample (DMSO, 400 MHz).

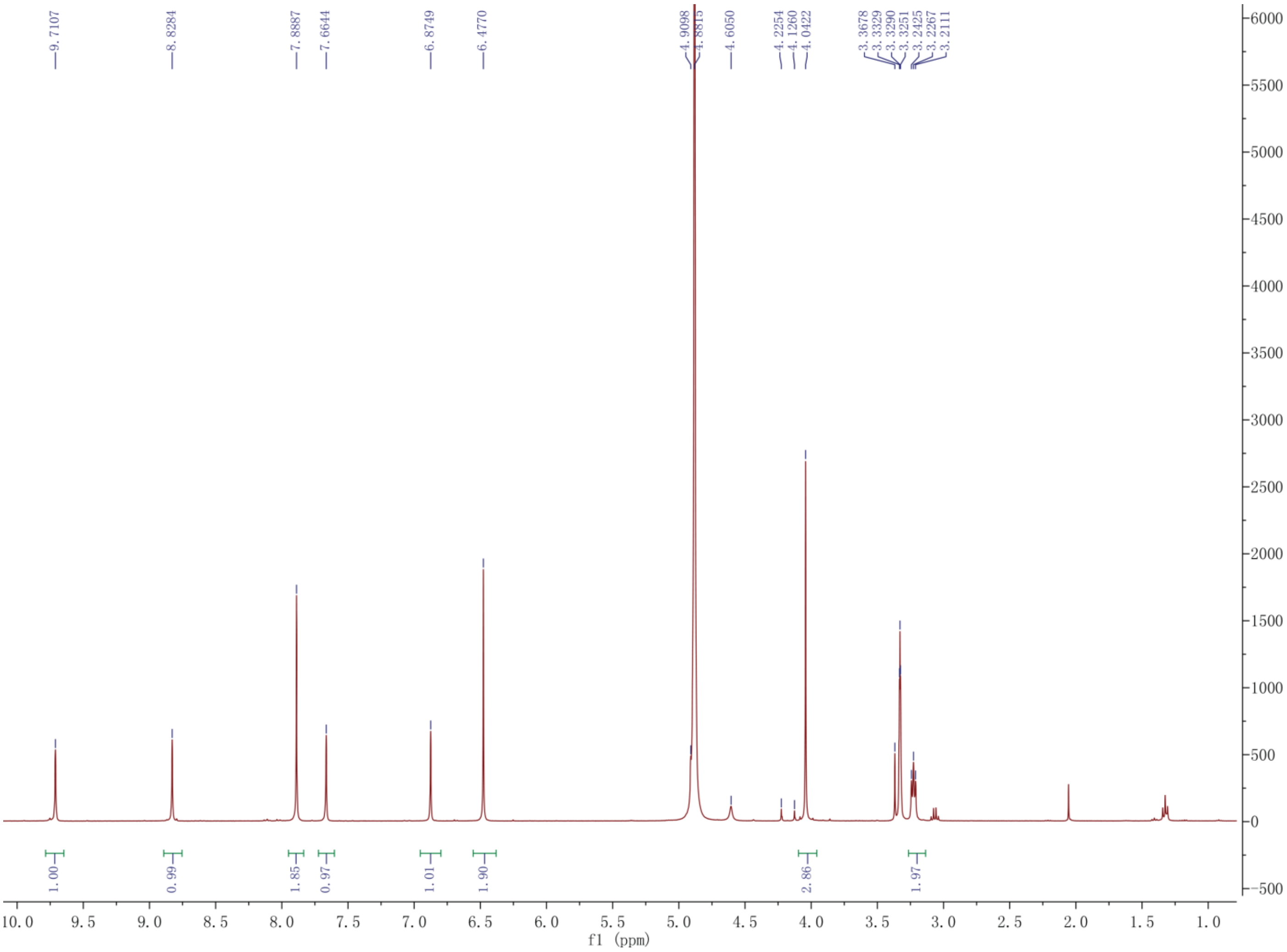

Figure S8. HPLC chromatogram of groenlandicine sample.

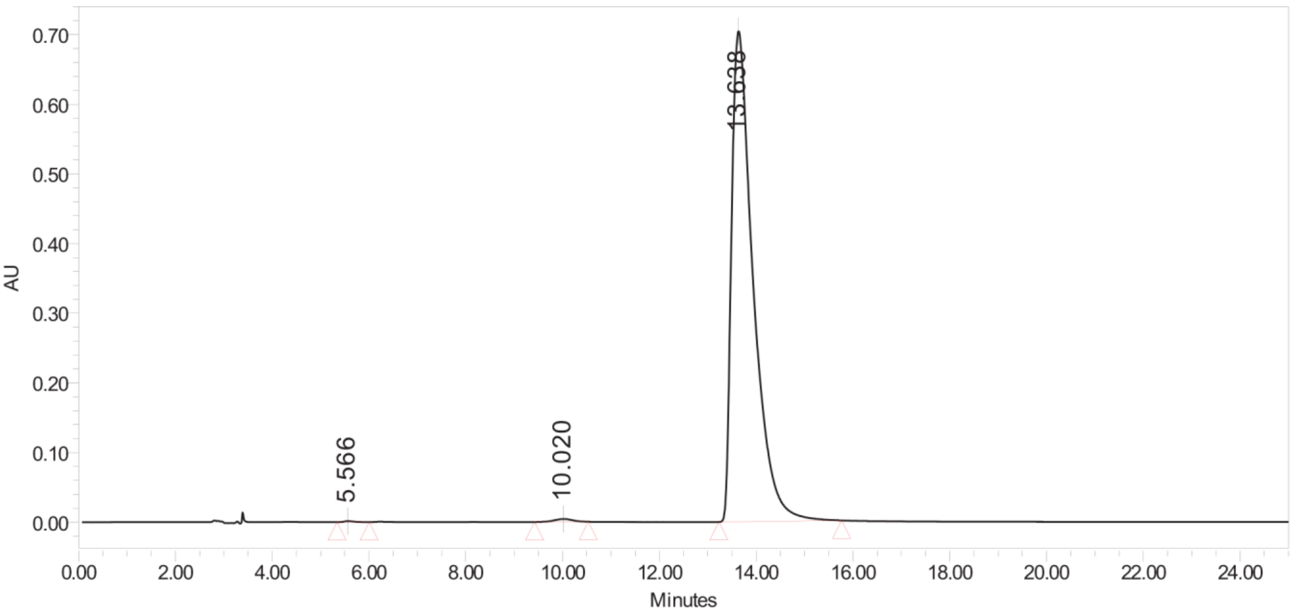

Peak Results

	Name	RT	Area	% Area	Height (μV)	USP Plate Count	USP Resolution
1		5.566	15585	0.07	1349	6431.80	
2		10.020	109327	0.50	4162	3486.01	8.80
3		13.638	21855645	99.43	703904	4671.35	4.73

Figure S9. 1H NMR spectrum of coptisine sample (DMSO, 400 MHz).

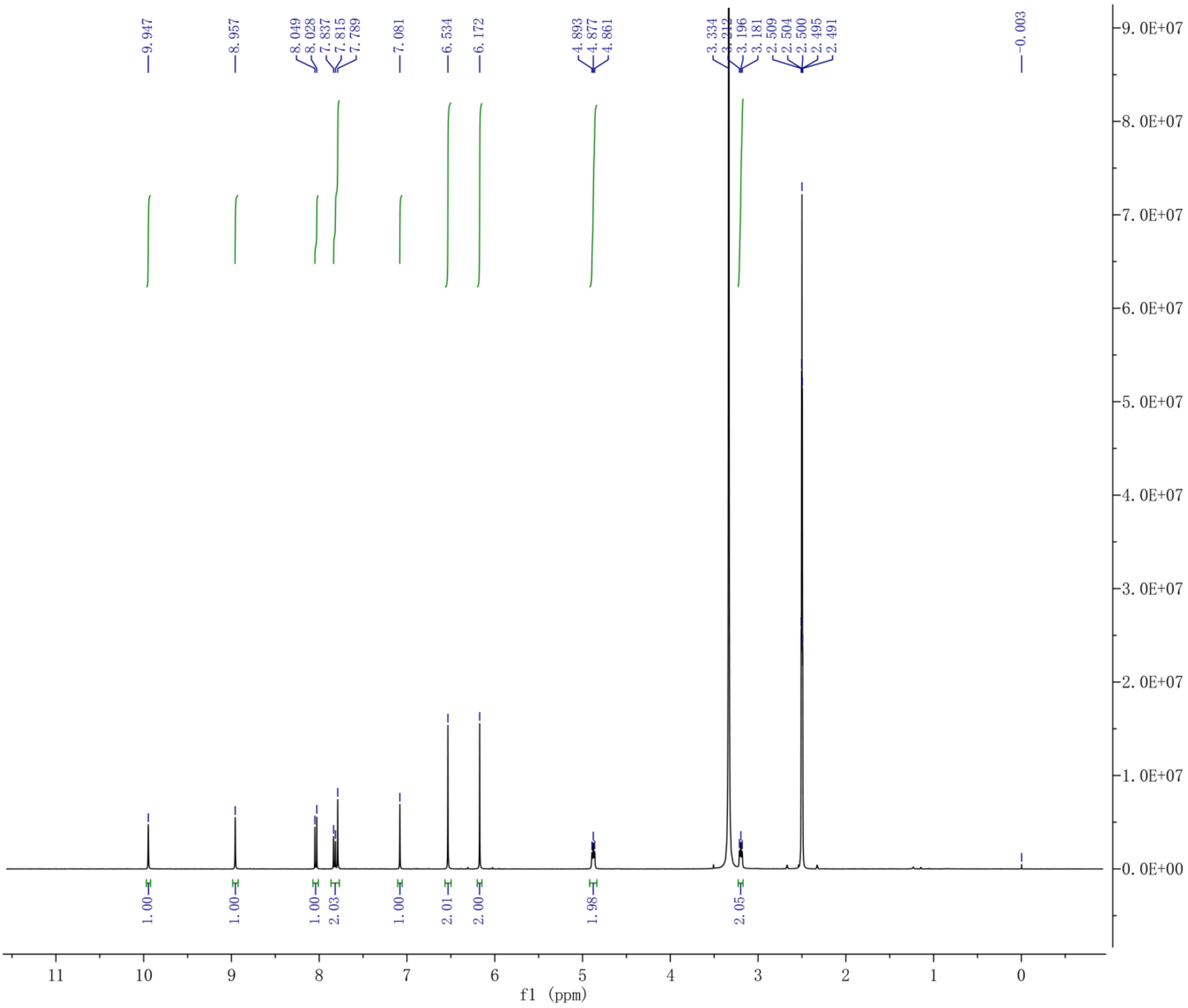

Figure S10. HPLC chromatogram of coptisine sample.

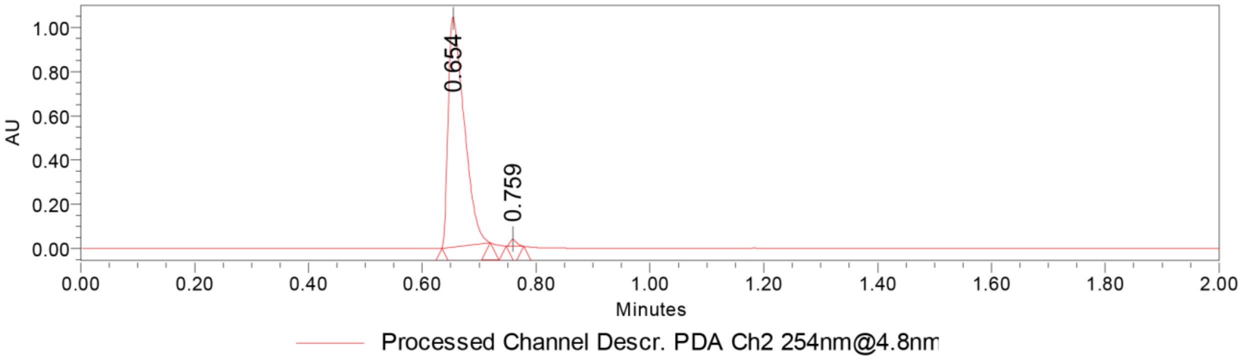

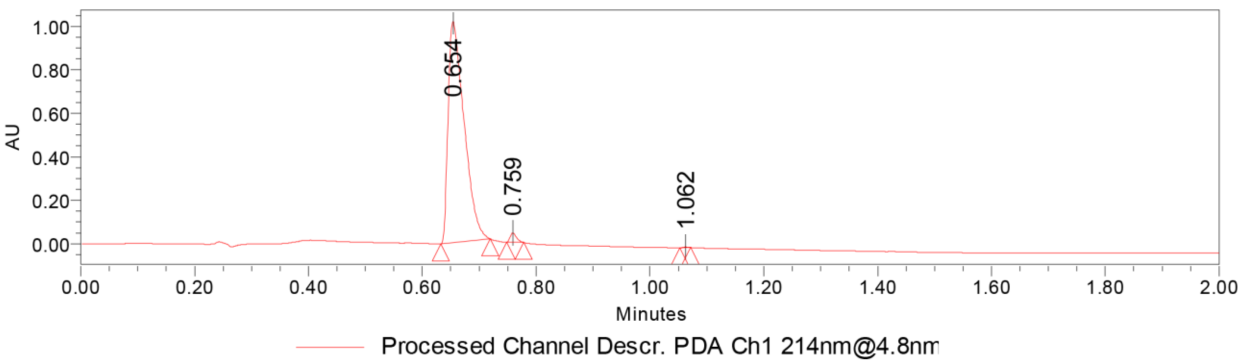

Peak Results
Channel Name: PDA Ch2 254nm@4.8nm

	RT	Width (sec)	Area	Height	% Area	Base Peak (Combined) (m/z)	Channel Name
1	0.654	5.100	2018431	1038936	98.82	158.32	PDA Ch2 254nm@4.8nm
2	0.759	1.850	24118	31296	1.18	318.36	PDA Ch2 254nm@4.8nm

Peak Results
Channel Name: PDA Ch1 214nm@4.8nm

	RT	Width (sec)	Area	Height	% Area	Base Peak (Combined) (m/z)	Channel Name
1	0.654	5.200	1978246	1016765	98.24	158.32	PDA Ch1 214nm@4.8nm
2	0.759	1.750	33248	43329	1.65	318.36	PDA Ch1 214nm@4.8nm
3	1.062	1.150	2091	3419	0.10	228.39	PDA Ch1 214nm@4.8nm

Figure S11. 1H NMR spectrum of berberine sample (DMSO, 400 MHz).

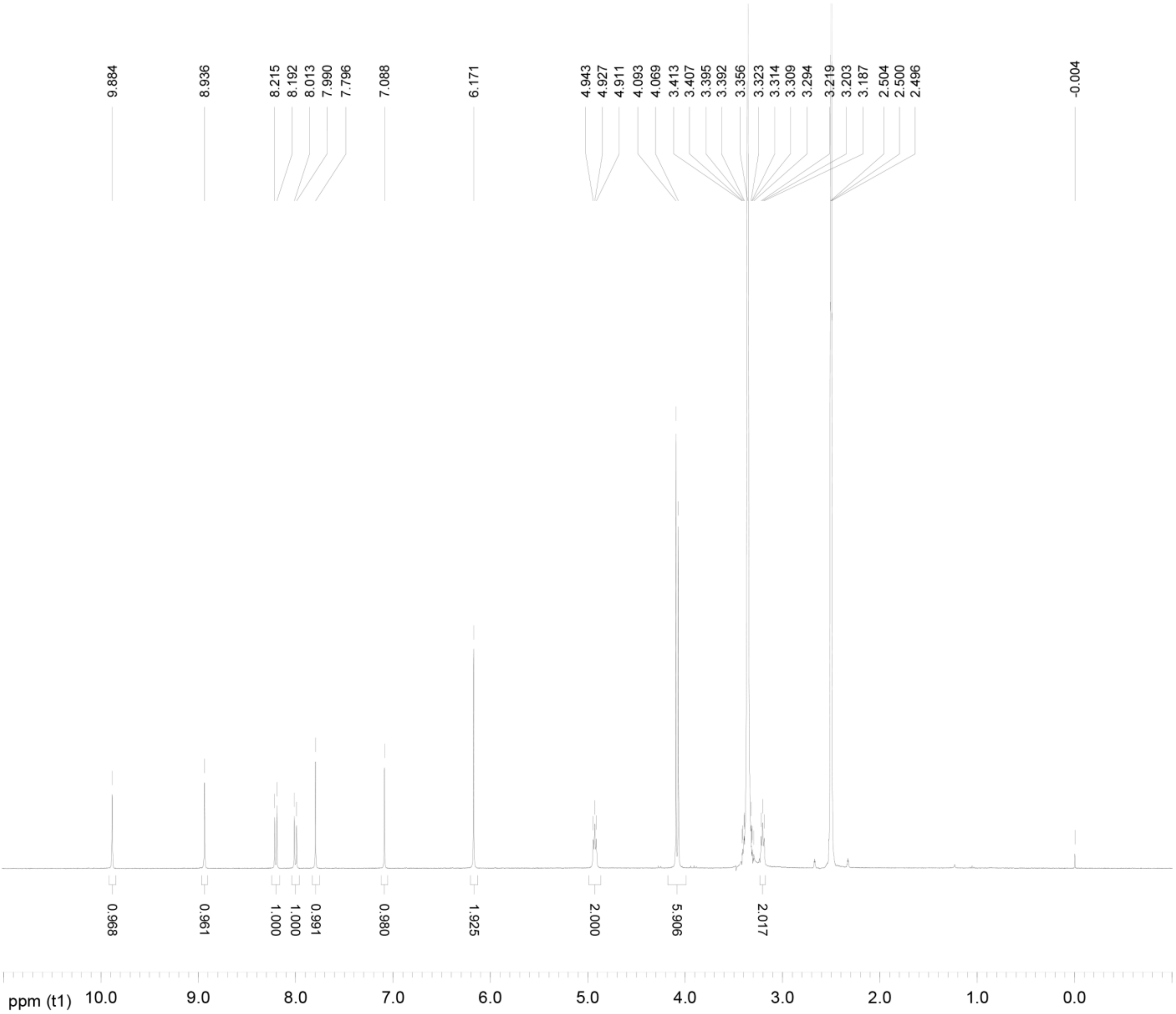

Figure S12. HPLC chromatogram of berberine sample.

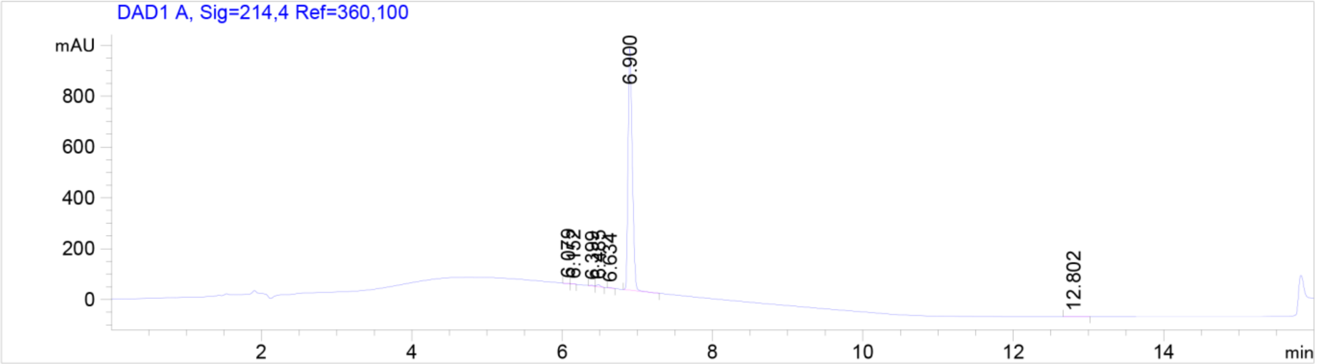

==

Area Percent Report

==

Sorted By : Signal
Multiplier: : 1.0000
Dilution: : 1.0000
Use Multiplier & Dilution Factor with ISTDs

Signal 1: DAD1 A, Sig=214,4 Ref=360,100

Peak #	RetTime [min]	Type	Width [min]	Area [mAU*s]	Height [mAU]	Area %
1	6.079	MF	0.0438	3.70940	1.41076	0.0976
2	6.152	FM	0.0432	2.61076	1.00777	0.0687
3	6.399	MF	0.0513	8.32024	2.70193	0.2189
4	6.485	FM	0.0542	27.83596	8.56259	0.7324
5	6.634	BB	0.0457	6.27722	2.31247	0.1652
6	6.900	BB	0.0599	3749.78467	953.51117	98.6570
7	12.802	BB	0.0845	2.29340	4.14630e-1	0.0603

Totals : 3800.83165 969.92131

==

*** End of Report ***

Figure S13. 1H NMR spectrum of palmatine sample (DMSO, 400 MHz).

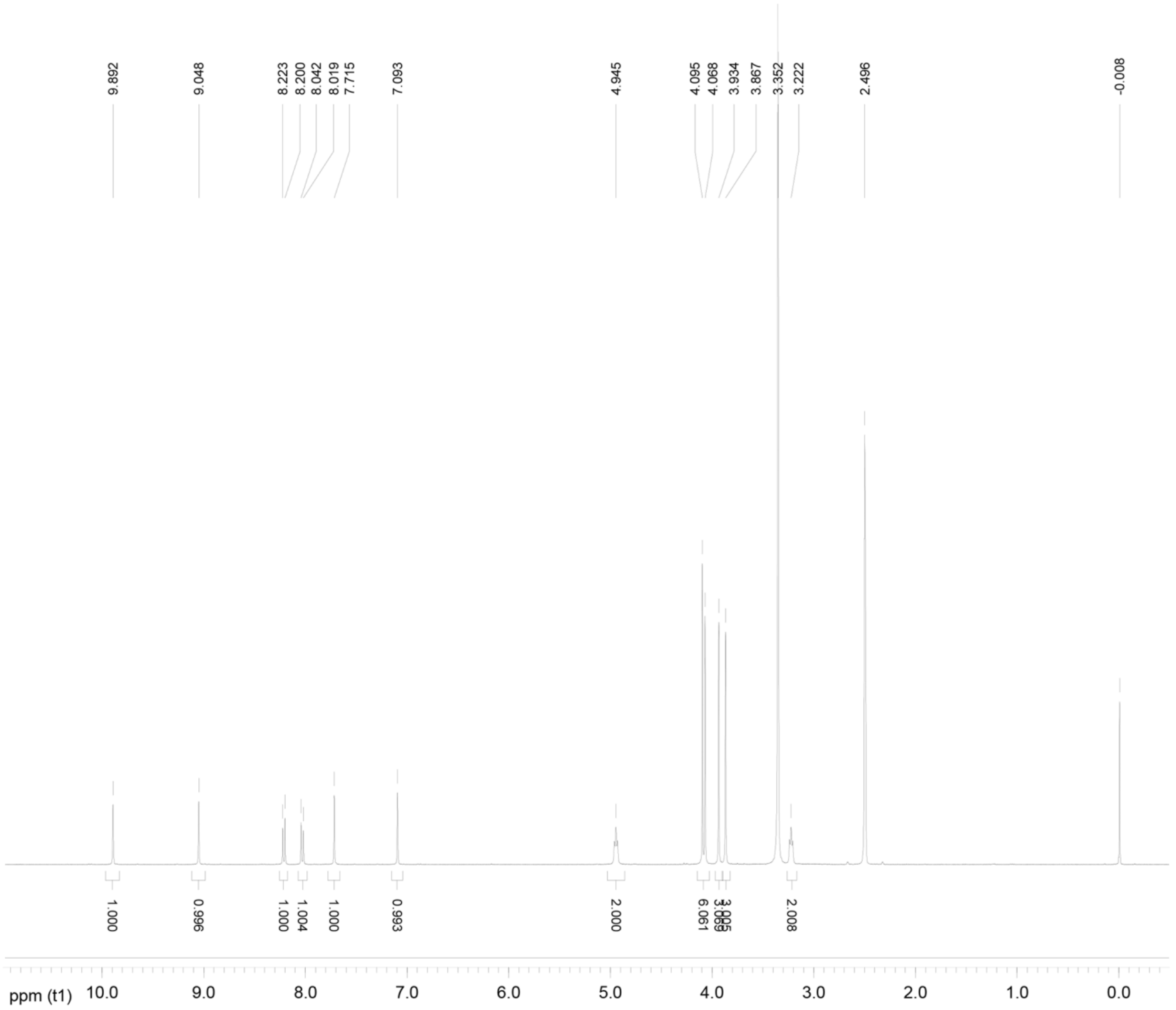

Figure S14. HPLC chromatogram of palmatine sample.

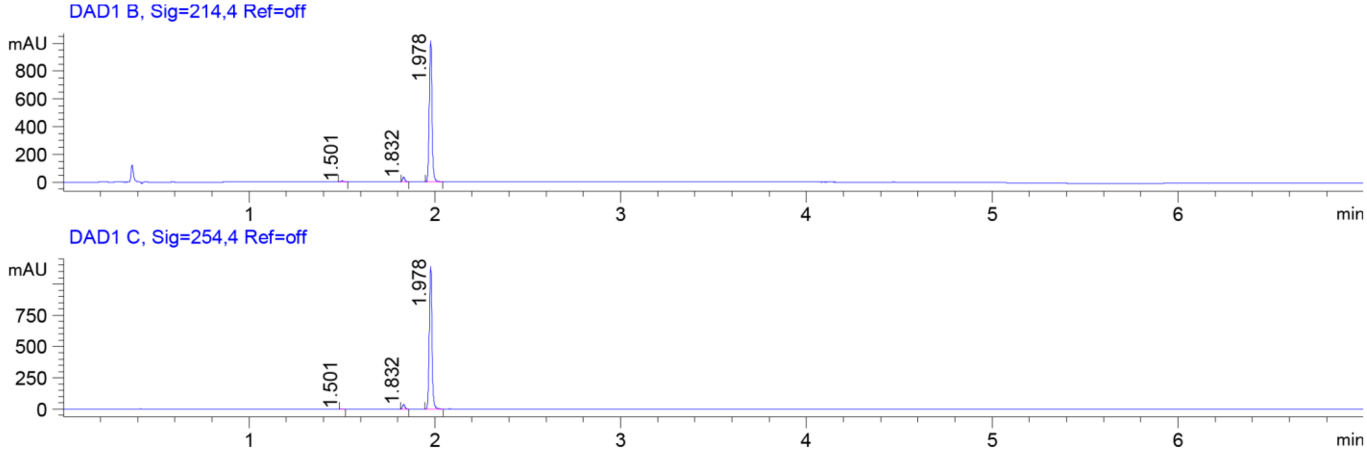

Integration Results for DAD1 B, Sig=214,4 Ref=off

RetTim	Width	Area	Height	Area%	MS(+)
1.50	0.02	9.38	8.50	0.87	ND
1.83	0.02	31.30	34.27	2.92	338
1.98	0.02	1031.99	1021.29	96.21	352

Integration Results for DAD1 C, Sig=254,4 Ref=off

RetTim	Width	Area	Height	Area%	MS(+)
1.50	0.02	2.11	2.00	0.18	ND
1.83	0.02	34.31	36.81	2.91	338
1.98	0.02	1141.40	1146.99	96.91	352

Figure S15. 1H NMR spectrum of jatrorrhizine sample (DMSO, 400 MHz).

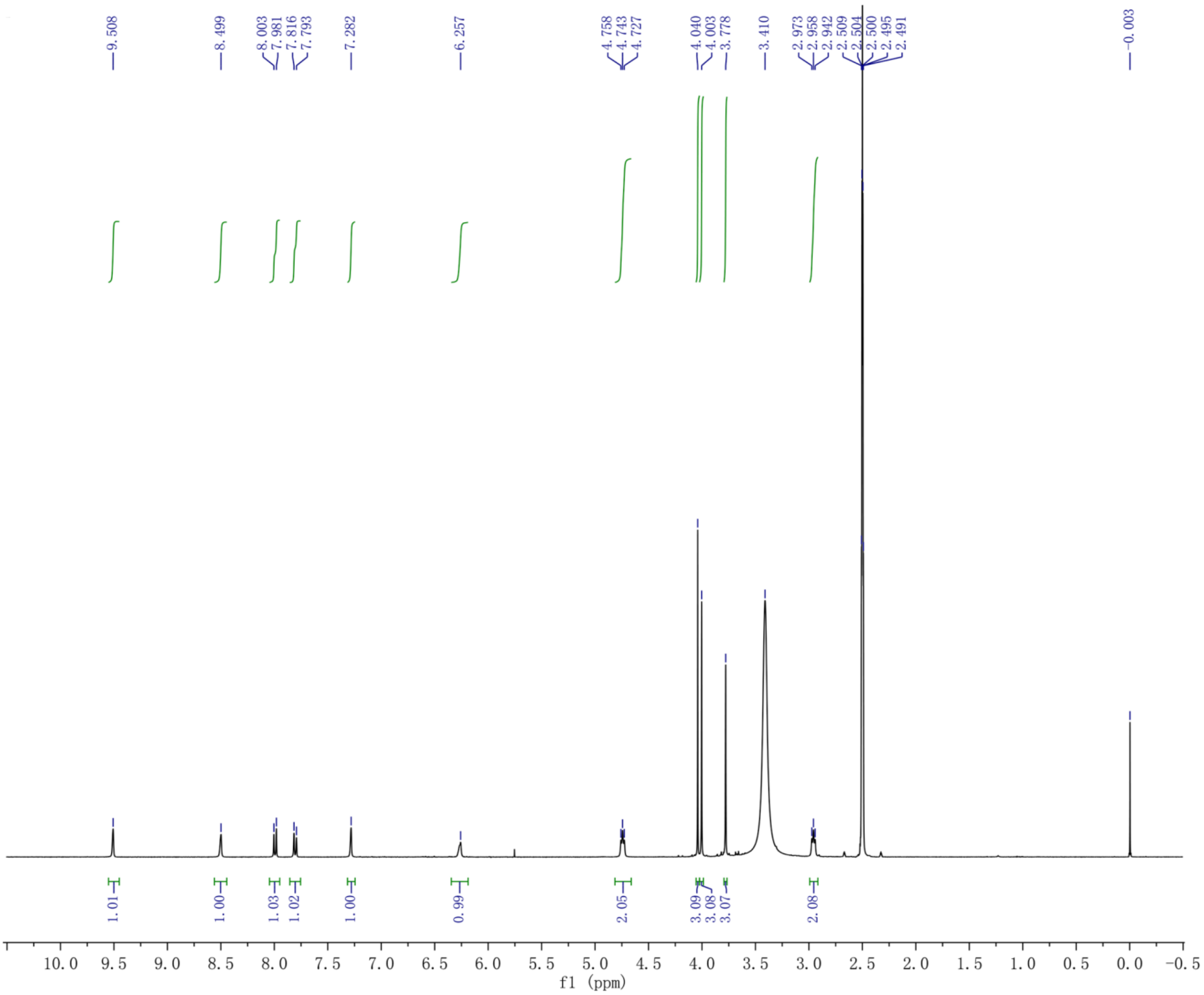

Figure S16. HPLC chromatogram of jatrorrhizine sample.

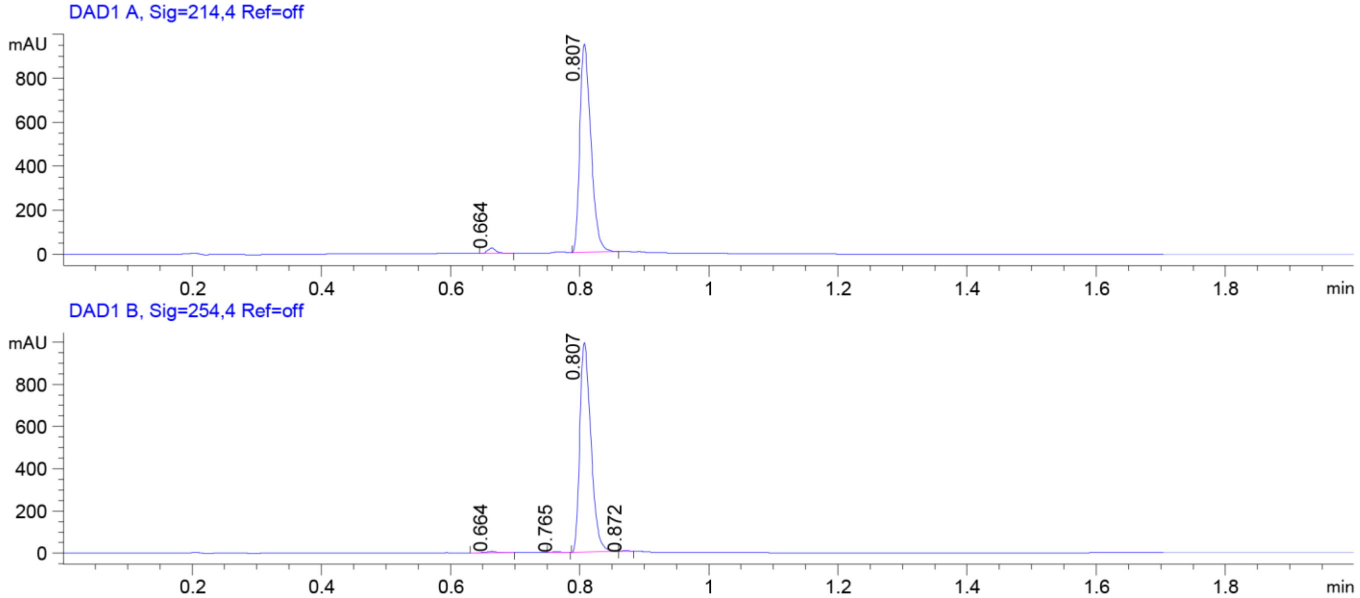

Integration Results for DAD1 A, Sig=214,4 Ref=off

RetTim	Width	Area	Height	Area%	MS(+)
0.66	0.01	22.07	25.23	1.93	338
0.81	0.02	1123.15	942.36	98.07	338

Integration Results for DAD1 B, Sig=254,4 Ref=off

RetTim	Width	Area	Height	Area%	MS(+)
0.66	0.01	5.40	5.89	0.45	338
0.76	0.01	3.33	4.05	0.28	338
0.81	0.02	1177.74	987.87	99.08	338
0.87	0.01	2.21	3.12	0.19	338

Figure S17. 1H NMR spectrum of berberrubine sample (DMSO, 400 MHz).

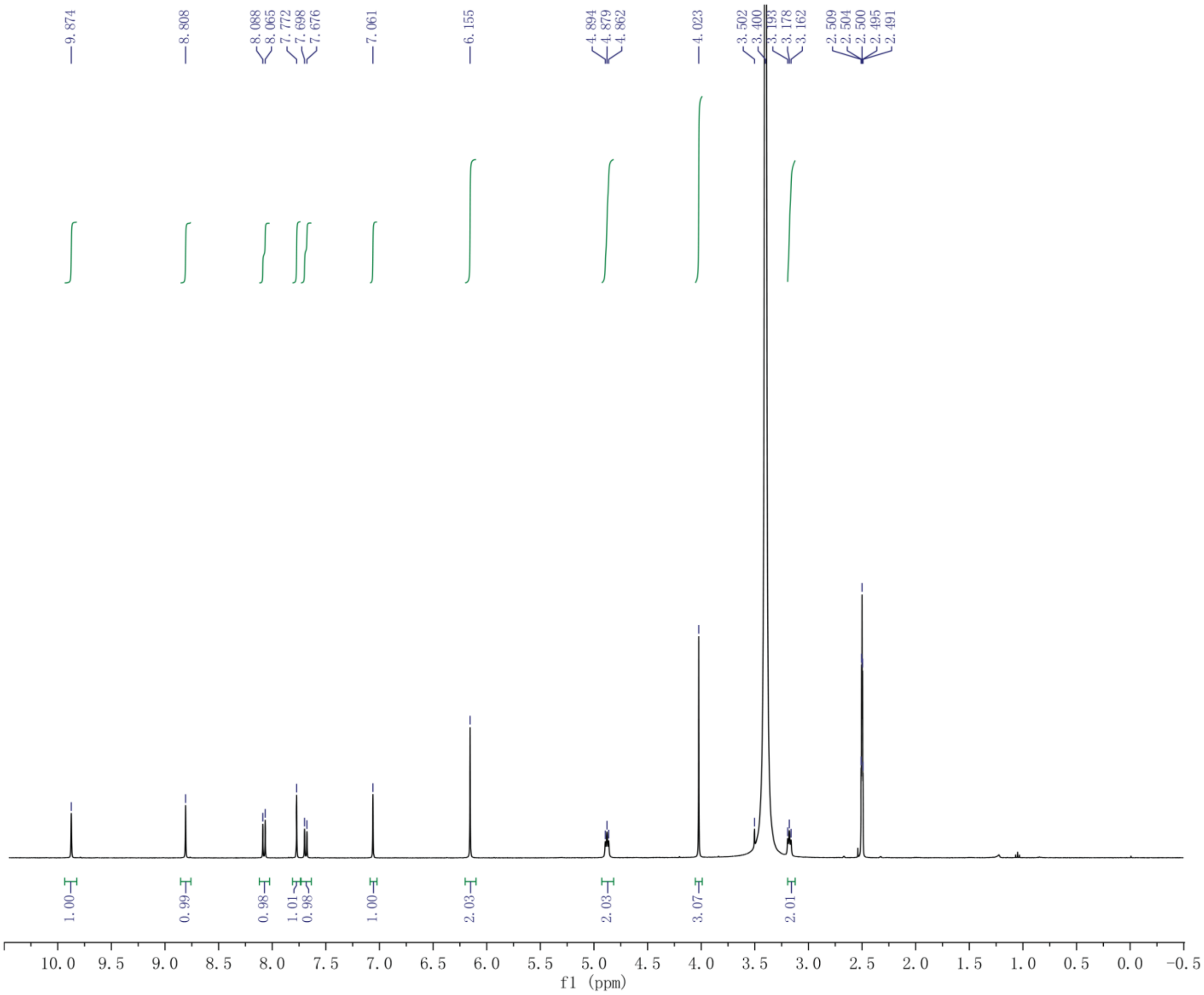

Figure S18. HPLC chromatogram of berberrubine sample.

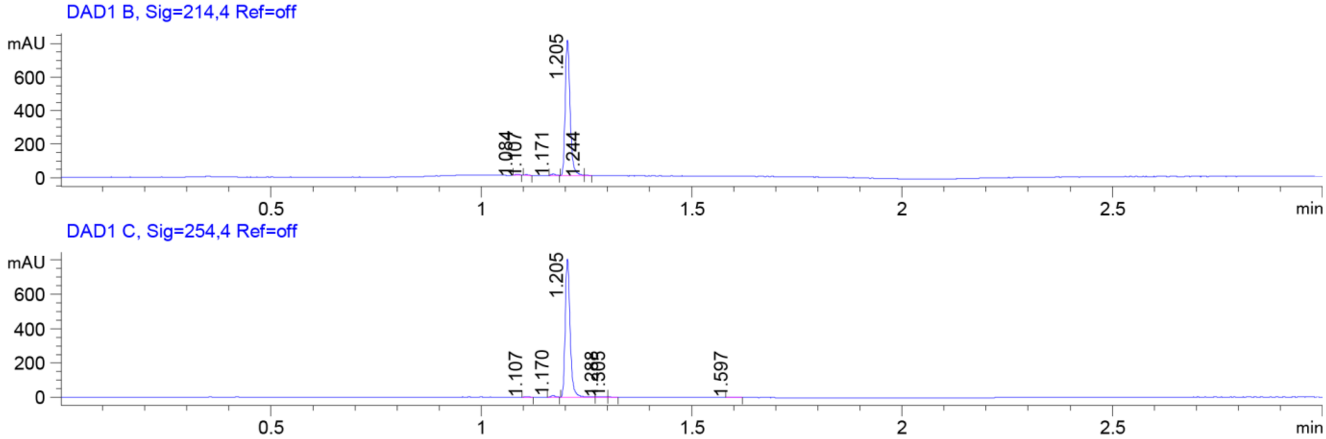

Integration Results for DAD1 B, Sig=214,4 Ref=off

RetTim	Width	Area	Height	Area%	MS(+)
1.08	0.01	3.35	4.61	0.51	209
1.11	0.01	2.65	4.01	0.40	209
1.17	0.01	5.27	7.51	0.80	308
1.20	0.01	645.95	809.50	98.02	322
1.24	0.01	1.80	3.31	0.27	322

Integration Results for DAD1 C, Sig=254,4 Ref=off

RetTim	Width	Area	Height	Area%	MS(+)
1.11	0.01	1.41	2.32	0.21	209
1.17	0.01	6.25	8.71	0.95	308
1.20	0.01	646.10	805.36	98.04	322
1.29	0.02	2.80	2.02	0.43	322
1.30	0.01	0.97	1.29	0.15	322
1.60	0.02	1.47	1.29	0.22	322

molecules

MDPI

Table S1. Summary of the information of the DPP-4 inhibitors discovered in this study.

Name	Structure	IC_{50} value of the DPP-4 inhibitory activity (μM)	K_D value of the DPP-4 binding assay (μM)	ΔG_{bind} value of the DPP-4-ligand systems	The interactions between DPP-4 and the ligand
columbamine	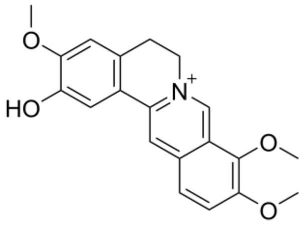	3.44	8.11	-31.84	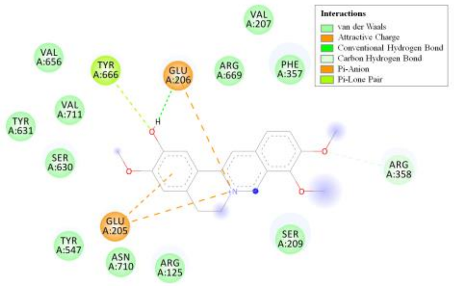
demethyleneberberine	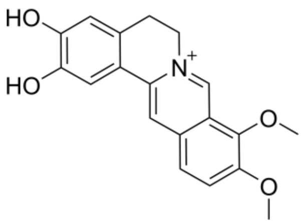	3.63	-	-28.73	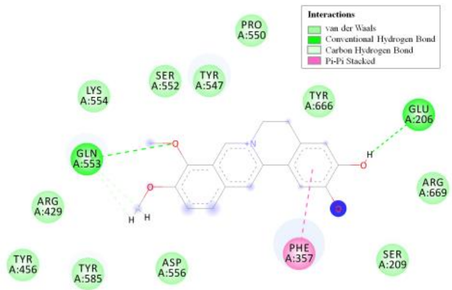

coptisine	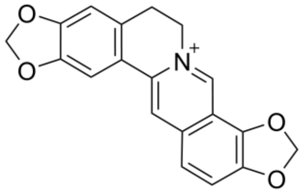	8.91	13.82	-17.98	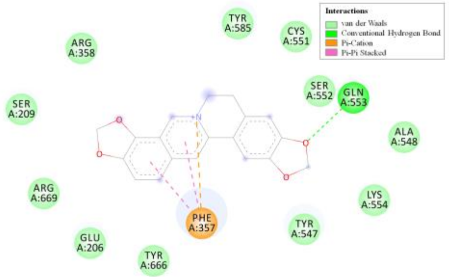
groenlandicine	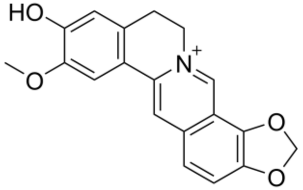	13.44	15.04	-19.42	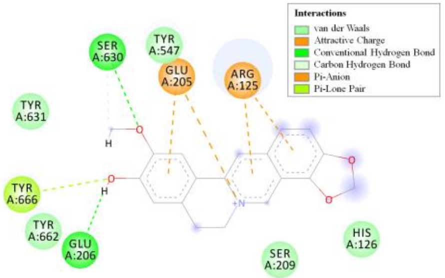
jatrorrhizine	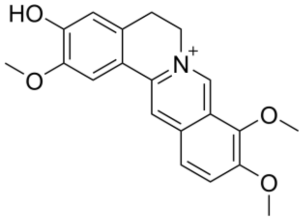	26.92	12.12	-26.97	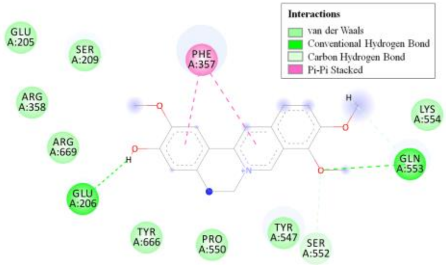

berberrubine	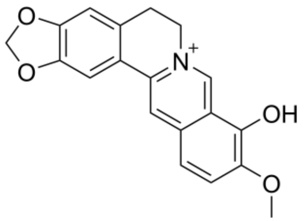	27.42	18.82	-26.75	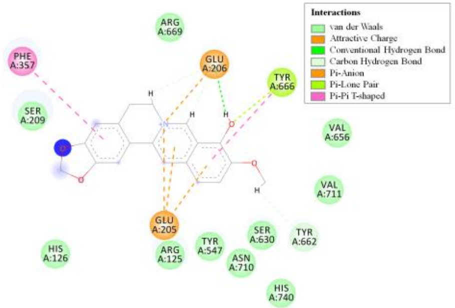
epiberberine	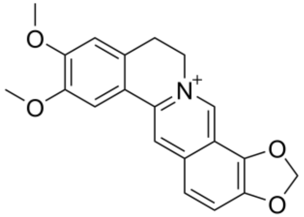	35.62	-	-16.06	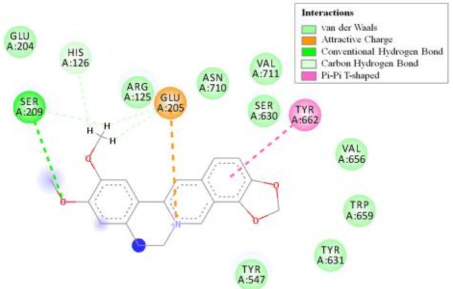
palmatine	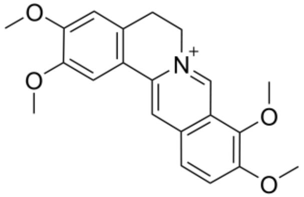	49.78	-	-24.08	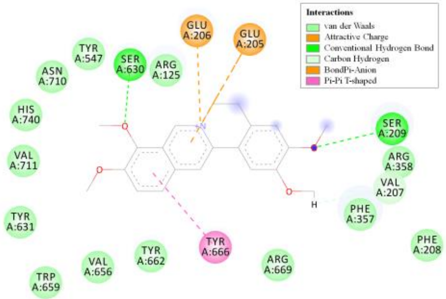

berberine	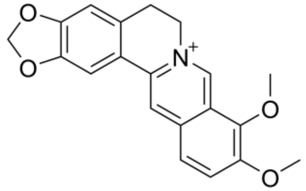	53.73	29.97	-19.72	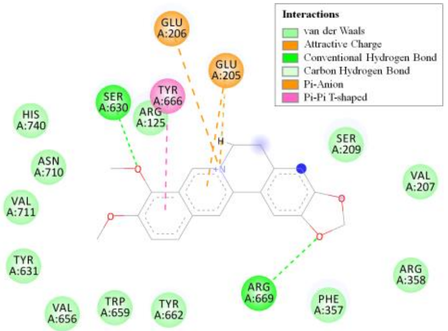
